# Multi-omics driven paradigm for construction of traditional Chinese Medicine *Zheng* (syndrome) diagnosis and treatment model, taking *Shi Zheng* (syndrome of dampness) as an example

**DOI:** 10.1186/s13020-025-01085-2

**Published:** 2025-03-08

**Authors:** Wenkai Wang, Le Yang, Wanhua Li, Ye Sun, Hui Sun, Yanjia Chen, Junling Ren, Jianwen Guo, Shuyun Wei, Fengye Lin, Guangli Yan, Ying Han, Qubo Chen, Xijun Wang

**Affiliations:** 1 State Key Laboratory of Dampness Syndrome of Chinese Medicine, The Second Affiliated Hospital of Guangzhou University of Chinese Medicine, Dade Road 111, Guangzhou, 510000 China; 2 State Key Laboratory of Integration and Innovation of Classic Formula and Modern Chinese Medicine, National Chinmedomics Research Center, Metabolomics Laboratory, Department of Pharmaceutical Analysis, Heilongjiang University of Chinese Medicine, Heping Road 24, Harbin, 150040 China; 3 Department of Neurology, Guangdong Provincial Hospital of Chinese Medicine, Guangzhou, 510000 China

**Keywords:** Metabolomics, Proteomics, SZ, TCM, Serum, Biomarkers, Pathological mechanism

## Abstract

**Background:**

*Shi Zheng* (SZ, syndrome of dampness) is a major syndrome type in traditional Chinese Medicine (TCM), the ambiguity of its pathomechanism and the lack of blood diagnostic indicators have limited the understanding of the development of SZ.

**Purpose:**

To explore the pathological mechanism of SZ and establish a symptom-centered diagnosis and treatment model.

**Methods:**

We recruited 250 participants, including healthy individuals and patients diagnosed with SZ. Serum metabolomics and proteomics analyses were performed to screen common pathways. Along with the biological significance of common pathways, a common pathway-symptom correlation diagram was constructed to elucidate the pathological mechanism underlying the occurrence and development of SZ. The enrichment score and correlations with SZ main symptom was used to screen the key common pathways. The key common pathways related to differential metabolites and proteins were used to establish a multi-index diagnostic model and protein therapy target group.

**Results:**

Joint metabolomics and proteomics analyses revealed 18 common pathways associated with symptoms. Six key pathways, such as pathogenic Escherichia coli infection, rheumatoid arthritis, PPAR signaling pathway, bile secretion, GnRH signaling pathway, and fat digestion and absorption were correlated with the main symptoms of SZ. These symptoms included greasy/thick/slippery tongue coating, heavy head, heavy body, heavy limbs, heavy joints, greasy hair, sticky mouth, sticky stool, and damp scrotum. Moreover, seven differential metabolites related to the key pathways were identified: LysoPA (20:3(5Z,8Z,11Z)/0:0), prostaglandin E2, leukotriene B4, lithocholate 3-O-glucuronide, 3-hydroxyquinine, lithocholic acid glycine conjugate, and PA(18:0/22:6(5Z,8E,10Z,13Z,15E,19Z)-2OH(7S, 17S)), and the combined diagnostic value of the seven indicators was the highest (discovery cohort: AUC = 0.90; validation cohort: AUC = 0.99). There were 23 differential proteins related to the key pathways, and six protein targets were identified, including RHOA, TNFSF13, PRKCD, APOA2, ATP1A1, and FABP1.

**Conclusion:**

The combined analysis of metabolomics and proteomics established a symptom-centered diagnosis and treatment model of *Shi Zheng*.

**Graphical abstract:**

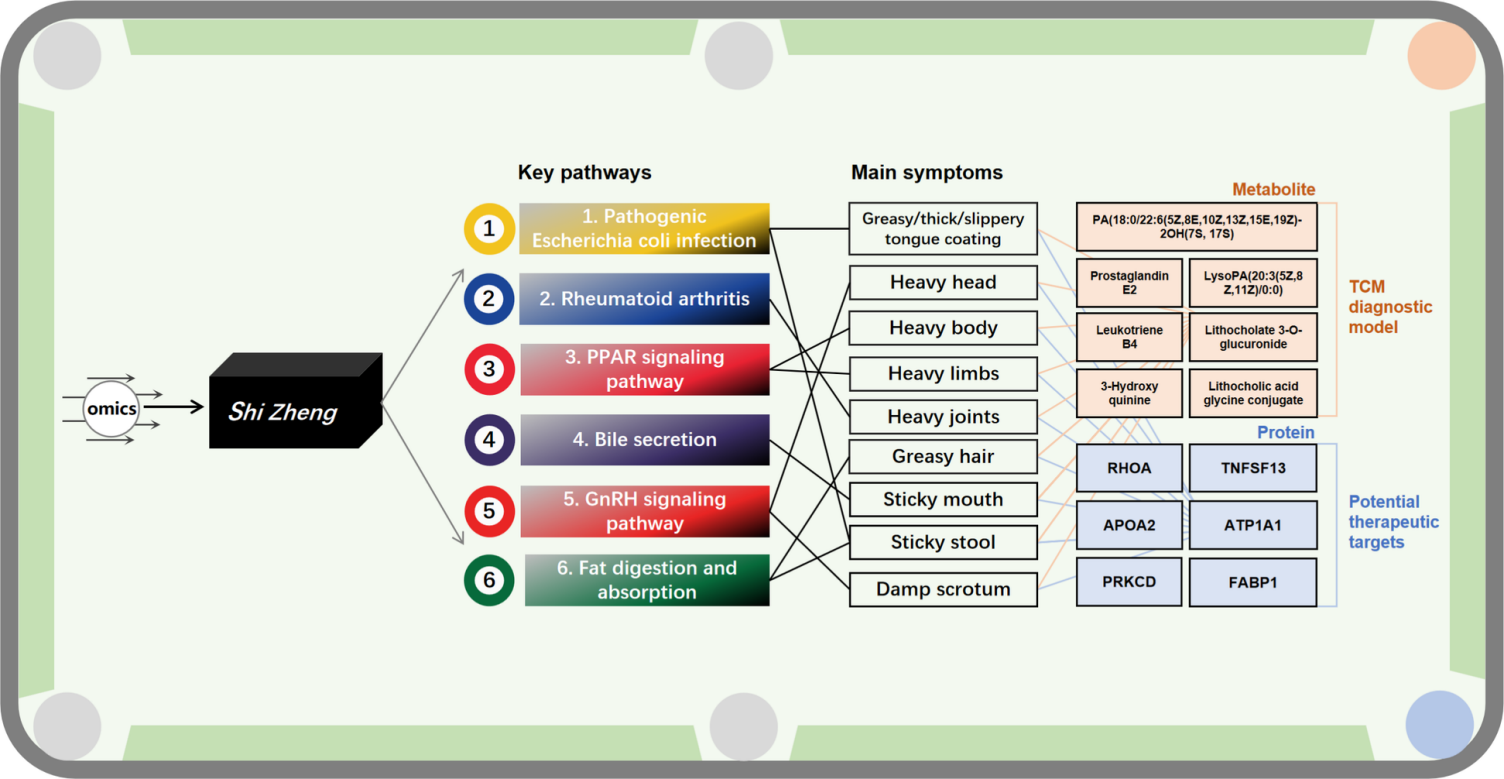

**Supplementary Information:**

The online version contains supplementary material available at 10.1186/s13020-025-01085-2.

## Background

“*Zheng*” is a complex pathological state of humans in traditional Chinese medicine (TCM). It is a syndrome of a comorbid state, which is a highly generalized and abstracted overall judgment of diseases based on the analysis of various symptoms in patients. *Zheng* characterizes pathological changes in patients at a certain stage. External pathogenic factors attack human organs, tissues, and blood, leading to abnormalities in the patient’s body and resulting in several symptoms. In the past, due to underdeveloped technology and incomplete knowledge of anatomy, doctors in ancient China were unable to observe pathological changes in diseases directly. However, by collecting the signs and symptoms of patients, combined with existing medical knowledge, they can predict the essence of pathological changes in the disease and call the predicted results “*Zheng*”. However, not all predicted results are correct. Over time, accurate and clinically valuable types of *Zheng* have been summarized. *Zheng* is equivalent to the simple assessment of the pathogenesis of diseases by Chinese doctors in the ancient and has extensive and complex characteristics. Although the biochemical and pathological diagnosis is relatively complete, several functional abnormalities cannot be detected through biochemical and pathological examinations, such as habitual dislocation, weakness, and subhealth status [1–3], but *Zheng* included these abnormalities. However, the diagnosis and treatment of *Zheng* rely on the experience of physicians and lack unified quantitative standards. Therefore, based on the common characteristics of *Zheng*, the establishment of diagnostic models and therapeutic targets with TCM characteristics can help solve the research problems associated with *Zheng*.

Some researchers used a single indicator to identify *Zheng* and obtained certain results [4, 5]. However, as the diversity of research samples increases, the diagnostic value of individual indicators is affected, which may be due to the complexity of *Zheng*. The emergence of systems biology has provided an option for solving this problem. Omics is a systems biology approach that focuses on the comprehensive study of a certain population of molecules in an organism. This research approach can overcome the limitations of traditional single-molecule research and provide a new perspective in the field of life science. It integrates a large amount of information to identify and assess the pathological and physiological states of patients and identify biomarkers related to *Zheng* in a large number of samples [6, 7]. Application of the omics method has shown promising results in research on *Zheng,* such as Yang Huang, spleen deficiency, kidney yin deficiency, and blood stasis [8–11]. This evidence suggests that the omics approach has provided insights into the biomarkers of *Zheng*. *Shi Zheng* (SZ, syndrome of dampness) in TCM is a general form of *Zheng* that causes many symptom changes due to disorders of water and liquid metabolism in TCM. The main symptoms include greasy tongue coating, thick tongue coating, slippery tongue coating, heavy head, heavy body, heavy limbs, heavy joints, greasy hair, sticky mouth, sticky stool, damp scrotum, and secondary symptoms are shown in Table 1 [12]. However, the ambiguity of the pathomechanism and the lack of blood diagnostic indicators have limited the development of SZ diagnosis and treatment. Therefore, in this study, we used a combination of metabolomics and proteomics to investigate the direct connection between common effector pathways and SZ symptoms. In addition, we focused on the main symptoms of SZ to obtain six key pathways, and innovatively designed a multi-indicator diagnostic model and a cluster of therapeutic targets corresponding to the main symptoms of SZ. This provides a reference for the diagnosis and treatment of SZ, and also explores new paradigm for the research of TCM *Zheng*.

**Table 1:** Reference table for diagnosis of SZ

	Symptoms
Specific indicators (main symptoms)	Greasy tongue coating, thick tongue coating, slippery tongue coating, heavy head, heavy body, heavy limbs, heavy joints, greasy hair, sticky mouth, sticky stool, damp scrotum
Sensitive indicators (secondary symptoms)	Obesity, dizziness, drowsiness, laziness and inactivity, inhibited sweating, dirty face, excessive phlegm, stuffy chest, lack of thirst, light and tasteless mouth, heavy mouth odor, lack of appetite, bloating in the abdomen, sloppy stool, soreness in the waist and knees, joint muscle soreness/pain, enlarged tongue, slippery pulse, soggy pulse, moderate pulse

Diagnostic principle of SZ is to meet 1 specific indicator or 3 sensitive indicators

## Methods

### Clinical subject inclusion

**Fig. 1:**
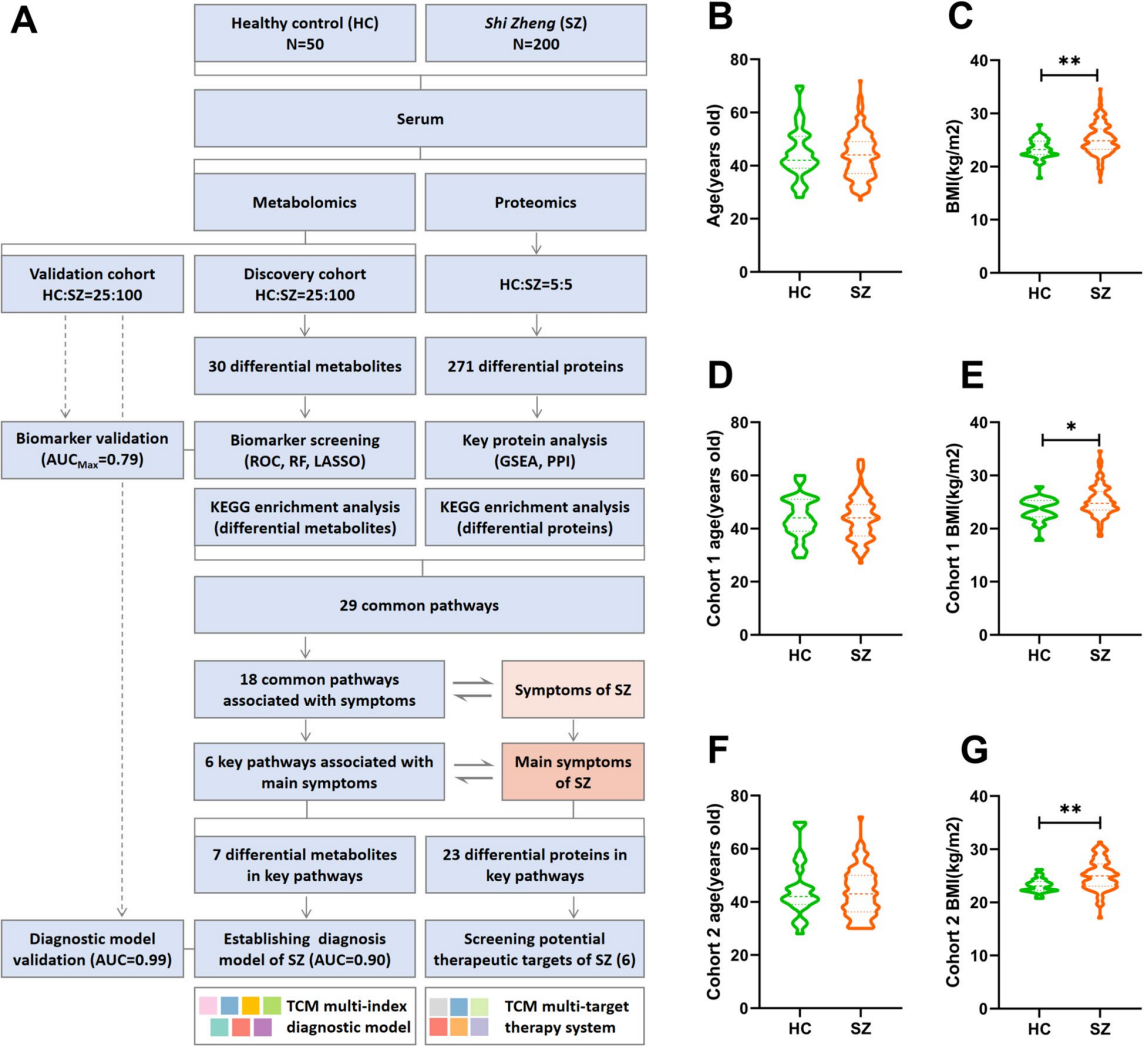
General information of clinical subjects. **A** Flow chart of omics analysis. **B**, **C** Comparison of age, BMI between HC (n = 50) and SZ (n = 200) groups. **D**, **E**: Comparison of age, BMI between HC (n = 25) and SZ (n = 100) groups in discovery cohort. **F**, **G**: Comparison of age, BMI between HC (n = 25) and SZ (n = 100) groups in validation cohort. Data were analyzed by Mann–Whitney U test, and the significant difference is indicated by *P* < 0.05, *P < 0.05, **P < 0.01

Two hundred male patients with SZ and 50 male healthy individuals who underwent physical examination in Guangzhou No. 11 People's Hospital from June 2022 to July 2022 were selected (Ethical code: K2022-03). There was no significant difference in age between the patients with SZ and the healthy individuals (Fig. 1A). Inclusion criteria for SZ: (a) Diagnosed as SZ by clinical physicians at Guangzhou No. 11 People's Hospital, and meet the diagnostic criteria of SZ [12]. The diagnostic table of SZ is shown in Table 1. (b) Exclude individuals with severe heart, liver, kidney, lung and other functional impairments, as well as those with acute and critical illnesses such as malignant tumors. (c) Exclude individuals with unclear consciousness and mental disorders. Inclusion criteria for healthy individuals: (a) The subjects stated that they have no obvious diseases, no obvious physiological abnormalities after physical examination, and does not meet the diagnostic criteria of SZ. (b) The subjects were diagnosed as healthy by clinicians at Guangzhou No. 11 People's Hospital. The study was approved by the Ethics Committee of Guangzhou No. 11 People's Hospital, and all participants completed case registration and signed informed consent. This study complied with the Declaration of Helsinki.

Randomly divide patients with SZ and healthy individuals into two cohorts using a random number table method: cohort 1 and cohort 2, with 25 healthy individuals and 100 patients with SZ in each cohort. Among them, cohort 1 is the discovery cohort for identifying potential biomarkers; Cohort 2 is an optimization and validation cohort that optimizes and validates the results of the discovery cohort. After grouping, there was still no significant difference in age between patients with SZ and healthy individuals within cohort 1 and cohort 2 (Fig. 1B, D, F).

### Sample collection

Blood was collected from the subjects and left undisturbed at room temperature for 40 min. Then, the samples were centrifuged at 4 °C and 4000 rpm for 15 min to separate the serum.

### Metabolomic analysis

#### Sample preparation

First, 50 µL of each serum sample was mixed with 200 µL of methanol, shaken for 1 min, and left undisturbed for 20 min. Then, the samples were centrifuged at 4 °C and 13,000 rpm for 15 min, and the supernatant was passed through a 0.22 µm filter membrane for UPLC-MS analysis.

#### UPLC-MS analysis

Metabolomic analysis was conducted with a UPLC instrument (Ultimate 3000, Thermo Scientific) and an Orbitrap analysis platform (Q-Empirical Focus, Thermo Scientific). The samples were prepared using a 100 mm ACQUITY UPLC HSS T3 chromatographic column (Waters, USA) and separated at 35 °C. The mobile phase was acetonitrile (A)–0.1% formic acid in water (B), and the UPLC gradient program was as follows: 0–1.0 min, 95–75% B; 1.0–2.0 min, 75–40% B; 2.0–7.5 min, 40–10% B; 7.5–10.5 min, 10–1% B; 10.5–12.5 min, 1% B; 12.5–13.0 min, 1–95% B; and 13.0–15.0 min, 1% B. The flow rate was 0.30 mL/min, and the temperature of the automatic sampler was maintained at 4 °C. After separation, mass spectrometry analysis was performed using a Q Exactive Focus instrument. Heater temperature, 320 °C; sheath gas flow rate, 40 arb; auxiliary gas flow rate, 15 arb; purge gas flow rate, 1 arb; positive ion spray voltage, 3.5 kV; negative ion spray voltage, 3.2 kV; capillary temperature, 350 °C; and S-Lens RF level, 50%. Scanning mode: First-level full scan (m/z 80–1200), resolution 70,000.

#### Metabolomic data analysis

In metabolomic analysis, the raw data was first preprocessed to remove ions with a value of 0 ≥ 50%. Principal component analysis (PCA) is an unsupervised data dimensionality reduction method, which converts high-dimensional data into low-dimensional data by linear transformation, while retaining the main features of the data as much as possible, which is used to determine the separation characteristics of the data. Orthogonal partial least squares discriminant analysis (OPLS-DA) is a supervised discriminant analysis method, which is suitable for the extraction of key features between two groups. These two methods are commonly used multivariate statistical analysis methods in metabonomics. Next, SIMCA 14.1 software was used to perform PCA on the metabolic data in cohort 1, and the model’s explanatory power and predictive accuracy were measured based on the values of the determination coefficient R^2^ and cross-validation coefficient Q^2^. To identify endogenous metabolites that play crucial roles in metabolic profile changes, OPLS-DA (supervised pattern recognition) was performed, and 200 permutation tests were performed to verify whether the patterns were overfitting. The rightmost point of the displacement test plots R^2^Y and Q^2^Y was always greater than all points on the left, and the intercept between the Q^2^ regression line and the Y-axis was less than 0, indicating a good fit. Based on the OPLS-DA model, the predicted VIP values were obtained, and the Mann–Whitney U test was conducted to calculate the P-values of the healthy group and the SZ group in cohort 1. Ions with VIP > 0.8 and *P* < 0.05 were selected as differential metabolites in cohort 1. Then, multivariate statistical analysis was conducted on the data of cohort 2 to identify overlapping ions in cohort 1 and cohort 2, and ions with inconsistently increasing or decreasing trends in the two cohorts were removed.

Endogenous screening was performed on the optimized ions, and then, based on the retention time (Rt) and mass–charge ratio (m/z) of key metabolites provided by UPLC-MS, element composition analysis (resolution greater than 20,000; precision less than 5 ppm) was performed to determine the possible molecular formula. Based on the exact mass, possible molecular formula, and secondary mass spectrometry (MS/MS) data, the Human Metabolome Database (HMDB, http://www.hmdb.ca) and other databases were searched to identify endogenous metabolites, and potential biomarkers of SZ were obtained. Next, we used cohort 2 as validation data, selected ROC curves to validate the diagnostic performance of potential biomarkers.

### Proteomic analysis

#### Sample preprocessing

Five serum samples were randomly selected from each of the SZ patients and healthy control. In total, 40 μL of 2.5% magnetic bead suspension was removed. After cleaning, the magnetic beads were resuspended in 100 μL of wash buffer. Then, 100 μL of serum was added, and the mixture was incubated at 37 °C and mixed evenly for 1 h. The supernatant was discarded by magnetic separation, and the mixture was washed with a wash buffer three times to obtain enriched serum protein. In total, 40 μL of the reductive alkylation and enzymatic hydrolysis system was added to the protein-enriched magnetic beads. After blowing and mixing, 1.2 μL of reductive alkylation reagent was added. After mixing, the mixture was heated at 95 °C for 5 min. After cooling to room temperature, 2 μL of equilibrium solution and 5 μL of enzymolysis solution were added, and the mixture was mixed well and subjected to enzymolysis at 37 °C for 2 h. After the termination reagent was added, the mixture was centrifuged at 20,000×*g* for 1 min, the supernatant was removed, a C18 column was used for peptide desalting, and the sample was concentrated using a vacuum freeze-centrifugation concentrator.

#### LC–MS/MS high-resolution mass spectrometry detection

A NanoDrop system was used to determine the peptide concentration of the samples. Before mass spectrometry injection, each sample was mixed according to the volume ratio of IRT:sample to be measured = 1:20 as the internal standard. The DIA liquid phase elution parameters were as follows: flow rate (400 nL/min), C18 analytical column (15 cm × 75 μm ID, 1.6 μm C18, ionopticks), buffer A (0.1% methanol aqueous solution), and buffer B (0.1% methanol acetonitrile). The elution gradient is shown in Supplementary Table 1. The scanning parameters of DIA mass spectrometry are as follows: capillary voltage (1.4 kV), dry temperature (180 °C), dry gas (3.0 L/min), mass range (100–1700 m/z), ion mobility (0.7–1.3), and collision energy (20–59 eV).

#### Data processing and analysis

The DIA raw data were processed using Spectronaut Pulsar 18.4 (Biognosys) software. The analysis module of the Spectronaut Pulsar software was opened, the parameter settings were set as per the software prompts (parameters are shown in Supplementary Table 2), and the quantitative data were exported after the analysis was completed. Differential proteins were screened based on the criteria *P* < 0.05 and FC < 0.83/FC > 1.25, and the Ouyi cloud website (https://cloud.oebiotech.com/) was used for subsequent analysis.

### Joint analysis of metabolomics and proteomics

KEGG pathway enrichment of differential metabolites and differential proteins was performed to identify the intersection of common pathways, and previous studies were consulted to identify the relationships between common pathways and SZ symptoms. A metabolite/protein-common pathway-symptom relationship diagram was constructed to identify the mechanism underlying symptoms and the corresponding metabolites and proteins. The diagnostic symptoms of SZ were divided into two parts (Table 1), including the main symptoms and secondary symptoms. The main symptoms were the common manifestations of SZ, the secondary symptoms were individualized manifestation indicators. In this study, we identified the common features of SZ and screened biomarkers that can be used for diagnosis. Therefore, we optimized the common pathways based on the main symptoms to identify the key pathways that could represent the common features of SZ. Then, differential metabolites corresponding to key pathways were identified, and diagnostic models corresponding to the main symptoms were established. The differential proteins in each pathway were sorted according to the *P* value, and the target proteins of each key pathway were screened along with the KEGG pathway map to establish a target protein group corresponding to the symptoms (Fig. 1A). In addition, in the joint analysis, we took the pathway enrichment results of differential metabolites and differential proteins as the starting point, and then gradually screened the key pathways, so the metabolic data and proteomics basically retained the original features. Also, when it comes to direct association analysis of metabonomic and proteomic content data, such as correlation analysis, only subjects with both types of data were selected.

### Statistical methods

The experimental data were analyzed using IBM SPSS Statistics 25 software. Unpaired t-tests were performed for the clinical observation of two groups of normally distributed data, and the data were expressed as the mean ± SD. The Mann–Whitney U test was conducted for the nonparametric test, and the data were expressed as the median and interquartile ranges.

## Results

### Physical characteristics of the clinical subjects

We divided healthy controls and SZ patients into two cohorts. Cohort 1 was the discovery cohort and was used for biomarker screening, while cohort 2 was the validation cohort for internal validation of biomarkers. First, we compared the age, BMI of the healthy control (HC) and SZ groups before they were split into cohorts. The results revealed no significant difference in age between the HC and SZ groups (Fig. 1B). The BMI in the SZ group were significantly greater than those in the healthy group (*P* < 0.01) (Fig. 1C). After the data were split into two cohorts, no significant difference in age between the SZ and HC groups in cohorts 1 and 2 was found, and the BMI maintained their original trends, indicating that the grouping was relatively random and did not change the original distribution of the data (Fig. 1B–G). Additionally, studies have shown a strong correlation between obesity and SZ [13], and obesity can cause abnormal water metabolism. The increase in BMI in the SZ group also supported this finding.

### Hexacosanoyl carnitine, leukotriene B4, prostaglandin E2, and other 30 substances were identified as differential metabolites of SZ

**Fig. 2:**
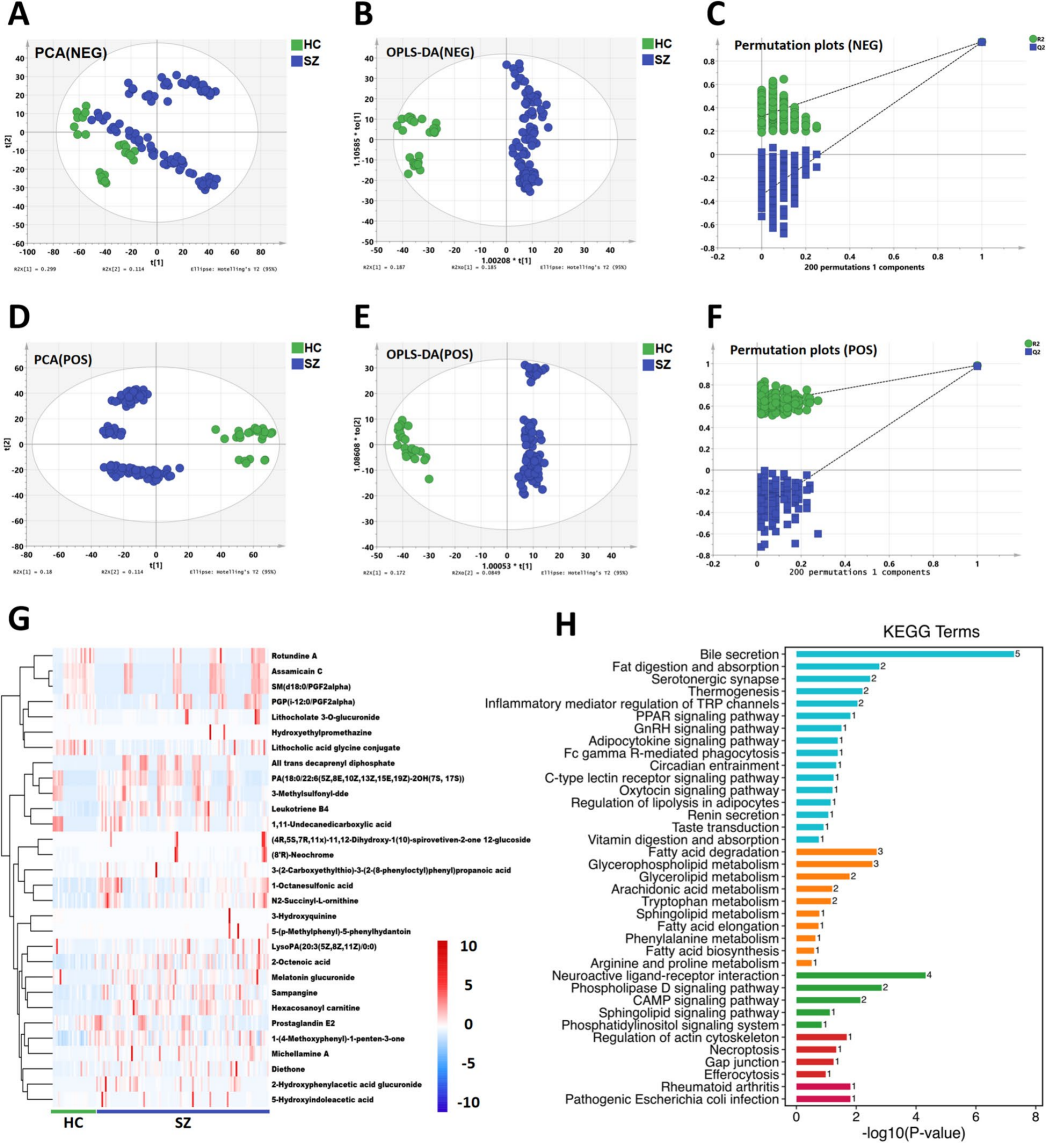
Screening and enrichment analysis of differential metabolite. **A**–**C** PCA, OPLS-DA scatter plots, and permutation plots of negative ions showed that the metabolic data of healthy controls and SZ patients had good separation characteristics. **D**–**F** PCA, OPLS-DA scatter plots and permutation plots of positive ions showed that the metabolic data of healthy controls and SZ patients had good separation characteristics. **G** Heat map of 30 endogenous differential metabolites. **H** KEGG enrichment analysis map of 30 differential metabolites

After UPLC-MS analysis and data preprocessing, 3302 negative ions and 5302 positive ions were obtained from cohort 1. We first conducted principal component analysis (PCA) on the metabolic data of the HC and SZ individuals in cohort 1. The PCA scatter plot (Fig. 2A, D) in the negative and positive ion modes showed good separation characteristics. Next, we conducted supervised pattern recognition OPLS-DA (NEG: R^2^ = 0.966, Q^2^ = 0.963; POS: R^2^ = 0.983, Q^2^ = 0.975). The OPLS-DA scatter plot (Fig. 2B, E) in the negative and positive ion modes showed good separation characteristics, and 200 response sorting tests also revealed that the models under the two ion modes (Fig. 2C, F) did not show overfitting. Next, VIP prediction values were obtained based on the OPLS-DA model, and VIP > 0.8 and *P* < 0.05 were used as screening conditions to obtain 4141 differential ions (1608 negative ions and 2533 positive ions).

To avoid false positives in subsequent validation (although there is a significant difference in the increasing or decreasing trend of differential metabolites in the two cohorts,, which still shows good diagnostic value), we removed ions with inconsistent average increasing or decreasing trends between the HC and SZ groups and obtained 35 overlapping ions. The ion chemical formula and name were determined based on the retention time (Rt) and mass–charge ratio (m/z) of overlapping ions, as well as, secondary mass spectrometry (MS/MS) data, and the Human Metabolome Database (HMDB) was searched (http://www.hmdb.ca). By matching the metabolic products with the mass spectrometry database, 30 endogenous differential metabolites were identified (Fig. 2G) (Supplementary Table 3). Subsequently, KEGG enrichment analysis was performed on the 30 different metabolites. The results revealed that SZ was mainly involved in five categories, including metabolism, environmental information processing, cellular processes, organismal systems, and human diseases (Fig. 2H).

### 1-Octanesulfonic acid, N2-succinyl-l-ornithine, and hexacosanoyl carnitine were selected as potential diagnostic indicators of SZ

The ROC curve is a common tool used to evaluate the performance of classification models. It uses the AUC to predict diagnostic value, but this method does not change with the changes in the distribution of categories. The random forest is a classifier that contains many decision trees. It can accurately classify the data and reveal important features. However, due to its randomness, the prediction results often fluctuate. LASSO regression can exclude unimportant features for modeling, effectively address multicollinearity problems, and provide a more stable model, but the number of selected features may be lower. All three methods have certain advantages and disadvantages. To screen more accurate and stable biomarkers, we simultaneously used the three methods to screen important features. The 30 differential metabolites were screened following the conditions of AUC > 0.8, *P* < 0.05, and 10 important metabolic characteristics were obtained (Supplementary Fig. 2A), which included hexacosanoyl carnitine, N2-succinyl-l-ornithine, 5-(*p*-methylphenyl)-5-phenylhydantoin, leukotriene B4, (4R,5S,7R,11x)-11,12-dihydroxy-1(10)-spirovetiven-2-one 12-glucoside, 1-octanesulfonic acid, stampangine, 2-octenoic acid, 3-hydroxyquinine, and lithocholic acid glycine conjugate. The results of random forest analysis revealed seven important features (Supplementary Fig. 2B), which included 1,11-undecanedicarboxylic acid, 3-hydroxyquinine, 1-octanesulfonic acid, 5-(*p*-methylphenyl-phenylhydantoin, PA(18:0/22:6(5Z,8E,10Z,13Z,15E,19Z)-2OH(7S, 17S)), N2-succinyl-l-ornithine, and hexacosanoyl carnitine. LASSO regression analysis revealed 12 features (Supplementary Fig. 2C, D), which included 1-octanesulfonic acid, leukotriene B4, N2-succinyl-l-ornithine, stampangine, 2-hydroxyphenylacetic acid glucuronide, diethone, all trans-decaprenyl diphosphate, 1-(4-methoxyphenyl)-1-penten-3-one, LysoPA (20:3(5Z,8Z,11Z)/0:0), hexacosanoyl carnitine, 2-octenoic acid, and melatonin glucuronide. Three potential biomarkers, 1-octanesulfonic acid, N2-succinyl-l-ornithine, and hexacosanoyl carnitine, were identified by the intersection of important features identified using the three methods (Supplementary Fig. 2E). The diagnostic value of the three potential biomarkers was subsequently verified in the validation cohort. The results of the ROC analysis revealed that the AUC of 1-octanesulfonic acid, N2-succinyl-l-ornithine, and hexacosanoyl carnitine was 0.76, 0.79, and 0.72; among them, the AUC of N2-succinyl-l-ornithine was the largest (Supplementary Fig. 2F).

### GAL3ST1, KPNB1, SNCA, and other 271 substances were identified as differential proteins of SZ

**Fig. 3:**
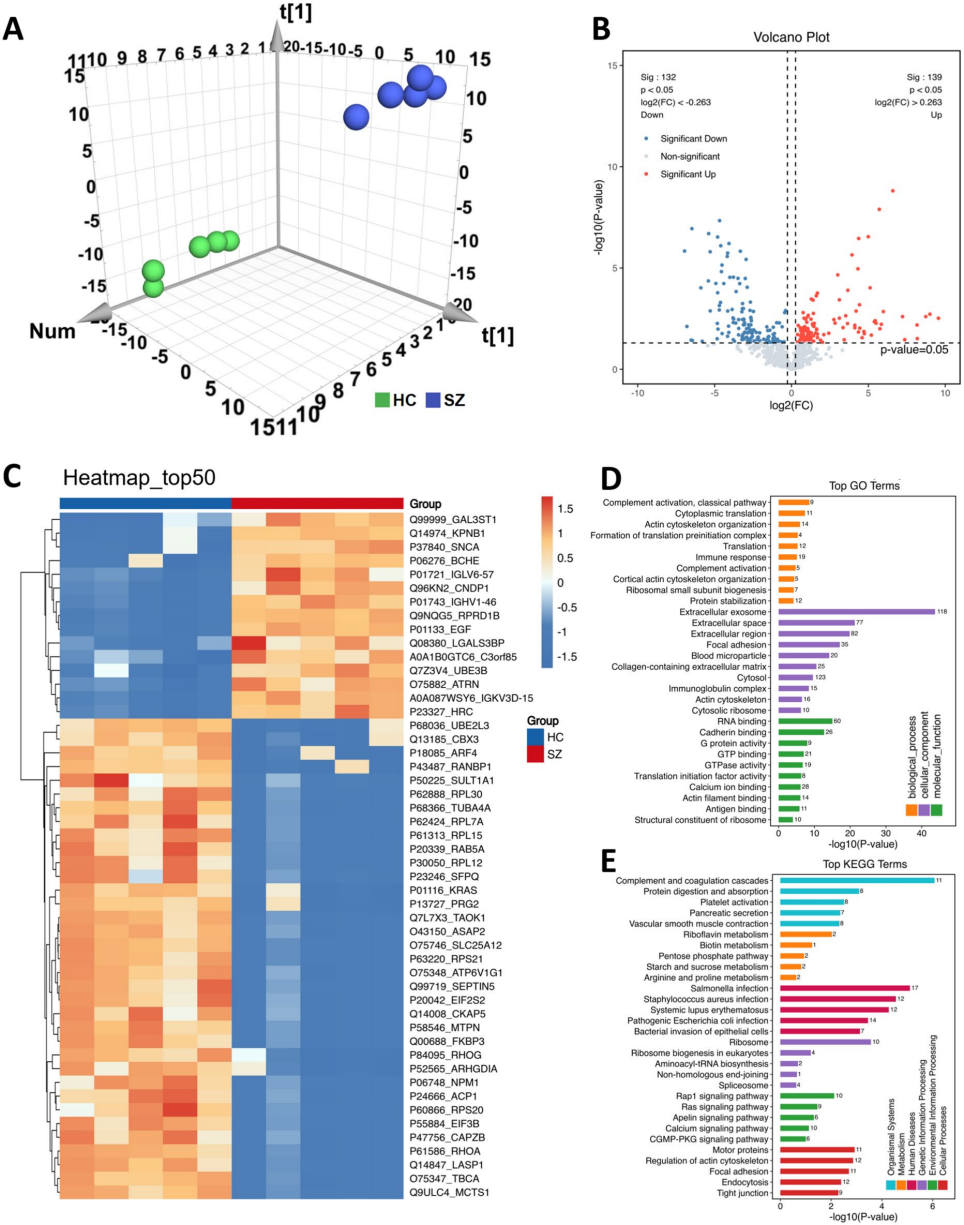
Screening and enrichment analysis of differential protein. **A** PCA 3D scatter plot showed that the protein data of healthy controls and SZ patients had good separation characteristics. **B** Volcano map of 271 differential protein screening. **C** Heatmap of top 50 differential proteins (*P* value). **D** GO enrichment analysis (The top 10 pathways are presented for each category). **E** KEGG enrichment analysis of 271 differential proteins (The top five pathways are presented for each category)

The proteomic analysis yielded 1066 protein features, all of which we imported into SIMCA to obtain a PCA 3D scatter plot (Fig. 3A), which revealed that the proteomic data of the HC and SZ groups had good separation characteristics. In total, 1066 proteins were screened based on the criteria of *P* < 0.05 and FC > 1.2/FC < 0.83 (Fig. 3B); 271 differential proteins were obtained, and the heat map of the protein content of the top 50 proteins (in ascending order of *P*-value) is shown in Fig. 3C. Differential proteins were analyzed for GO enrichment, and pathways in the three categories were sorted by *P*-value. The top 10 pathways are shown in Fig. 3D. Finally, KEGG enrichment analysis was performed on the differential proteins (Fig. 3E), and 252 pathways were obtained. The terms in each category are sorted according to the *P-*value. The top five terms of the organismal systems category include complement and coagulation cascades, protein digestion and absorption, platelet activation, pancreatic secretion, and vascular smooth muscle contraction. The top five terms in the metabolism category were riboflavin metabolism, biotin metabolism, the pentose phosphate pathway, starch and sucrose metabolism, and arginine and proline metabolism. The top five terms in the human diseases category were *Salmonella* infection, *Staphylococcus aureus* infection, systemic lupus erythematosus, pathogenic *Escherichia coli* infection, and bacterial invasion of epithelial cells. The top five terms in the genetic information processing category were ribosome, ribosome biogenesis in eukaryotes, aminoacyl-tRNA biosynthesis, nonhomologous end-joining, and spliceosome. The top five terms in the environmental information processing category were the Rap1 signaling pathway, Ras signaling pathway, Apelin signaling pathway, calcium signaling pathway, and cGMP-PKG signaling pathway. The top five terms in the cellular processes category were motor proteins, regulation of actin cytoskeleton, focal adhesion, endocytosis, and tight junction.

### HSPA5, KRAS, and RACK1 are the top three proteins in the mutual aid connectivity

**Fig. 4:**
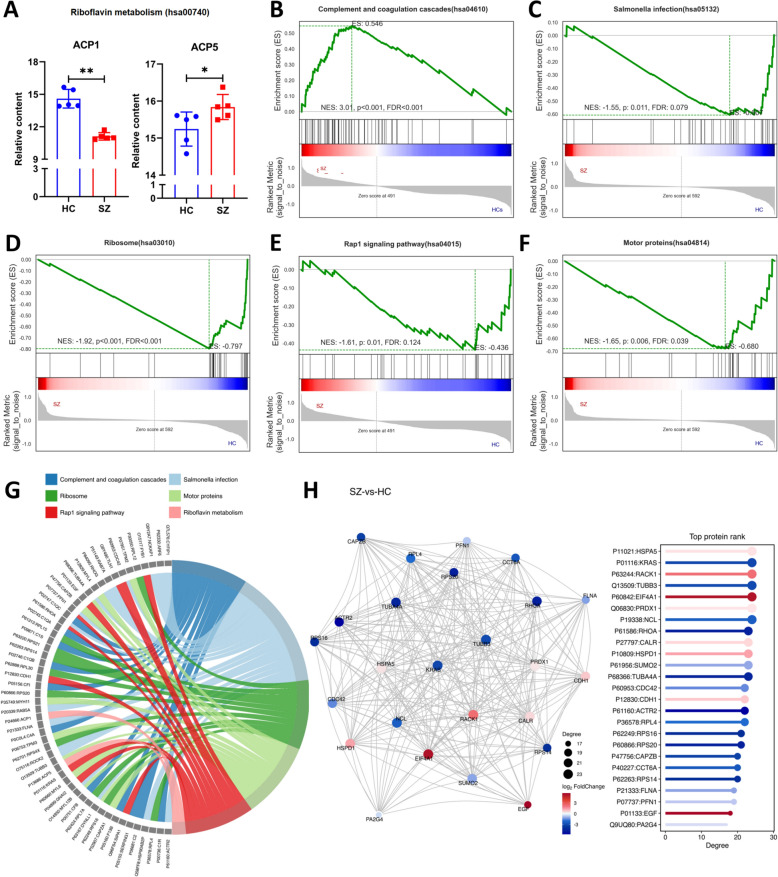
Analysis of GSEA in top-most term under each category and protein mutual aid network. **A** Comparison of protein content related to Riboflavin metabolism pathway in Metabolism category. **B** GSEA analysis revealed elevated expression of Complement and coagulation cascades pathway in Organismal Systems category of SZ patients. **C** GSEA analysis showed decreased expression of Salmonella infection pathway in Human Diseases of SZ patients. **D** GSEA analysis showed decreased expression of Ribosome pathway in Genetic Information Processing of SZ patients. **E** GSEA analysis showed decreased expression of Rap1 signaling pathway in Environmental Information Processing of SZ patients. **F** GSEA analysis showed decreased expression of Motor proteins pathway in Cellular Processes of SZ patients. **G** Chord diagram of top1 pathway in each category. **H** Top 25 connectivity protein interactions network map

We performed GSEA on the main terms (top-most term under each category) selected by KEGG. Among these pathways, the complement and coagulation cascades pathway was highly expressed in the SZ group (Fig. 4B), whereas the *Salmonella* infection pathway, ribosome pathway, Rap1 signaling pathway, and motor protein pathway were expressed at low levels in the SZ group (Fig. 4C–F). We could not perform GSEA of riboflavin metabolism because there were few related proteins in the pathway (Fig. 4A). Therefore, we compared the changes in the contents of ACP1 and ACP5 in this pathway. ACP1 in the SZ group was significantly lower than that in the HC group (*P* < 0.01), and ACP5 was significantly greater (*P* < 0.05).

Next, we used chordal diagrams to show the corresponding proteins of each major pathway (Fig. 4G), in which the pathways associated with complement and coagulation cascades corresponded to the proteins C1R, CFB, C1QA, C1QB, C1QC, SERPING1, CFI, F13B, C2, C1S, and C4A. The riboflavin metabolism pathway corresponded to the proteins ACP1 and ACP5. The *Salmonella* infection pathway corresponded to the proteins MYL12B, ROCK2, PFN1, HSP90AB2P, RAB5A, FLNA, RAB7A, CDC42, ACTR2, RHOA, ARF6, DYNLL1, TUBA4A, RHOG, TUBB3, CYFIP1, and NCKAP1. The ribosome pathway corresponded to the proteins RPL12, RPL4, RPS20, RPL15, RPS16, RPS14, RPL7A, RPS4X, RPL30, and RPS21. The Rap1 signaling pathway corresponded to the proteins FYB1, KRAS, EGF, GNAI2, PFN1, CDH1, CDC42, RHOA, SIPA1, and TLN1. The motor protein pathway corresponded to the proteins MYL12B, TPM3, TPM2, MYL4, MYH11, CAPZB, CAPZA1, MYL6, DYNLL1, TUBA4A, and TUBB3. Next, we analyzed the mutual aid relationships of different proteins and obtained a network diagram with the top 25 connected proteins (Fig. 4H).

### PPAR signaling pathway, taste transduction, oxytocin signaling pathway and other 18 common pathways are associated with SZ symptoms by joint analysis

**Fig. 5:**
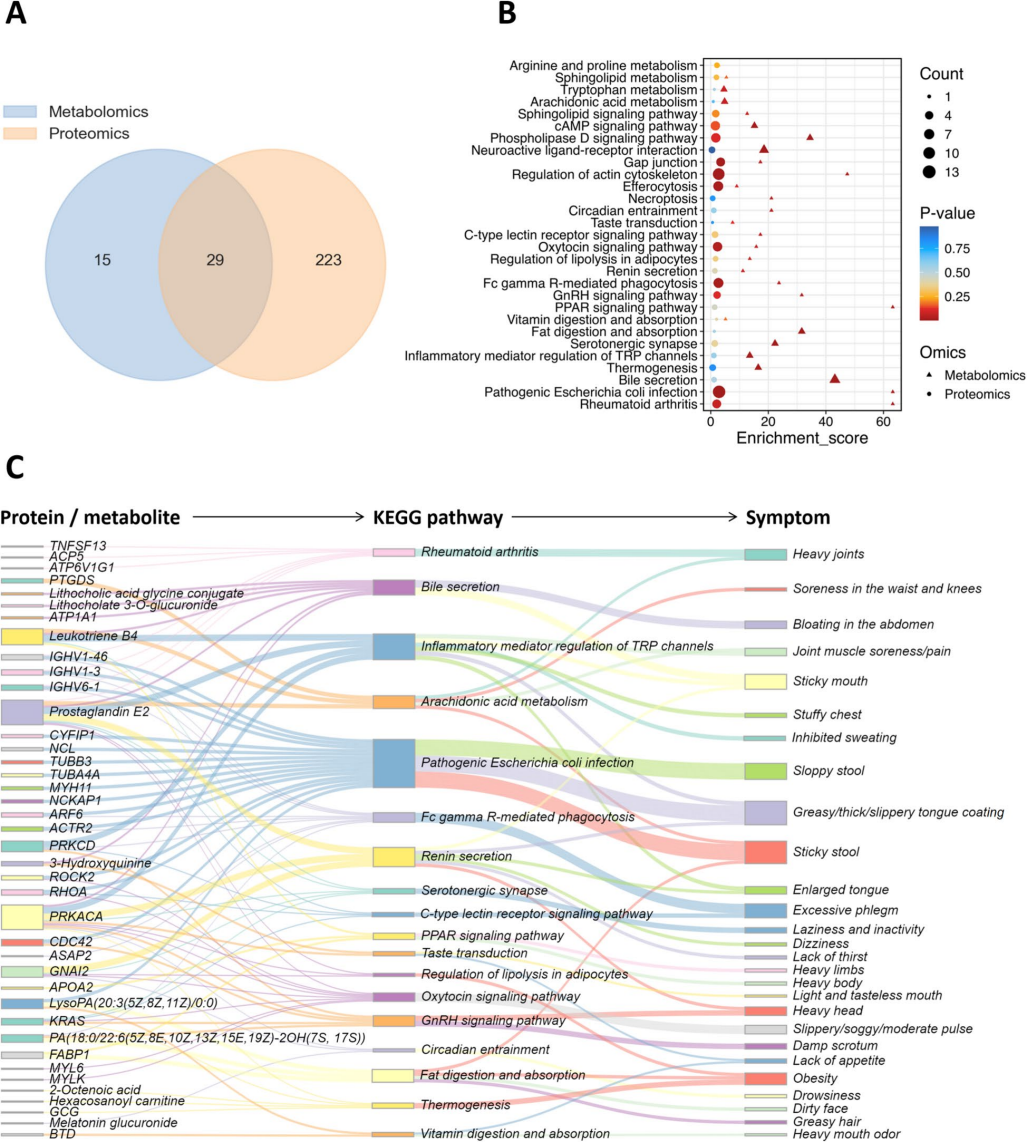
Analysis of metabolomics and proteomics common pathways and their association with SZ symptoms. **A** Venn plot of 29 pathways common to metabolomics and proteomics. **B** Bubble plot of the 29 common pathways. **C** Protein/metabolite-KEGG pathway-symptom association map screened out 18 common pathways (See supplementary Table 4 for references)

By analyzing the metabolomics and proteomics results, we identified the biological processes (KEGG pathways) related to the occurrence and development of SZ. To systematically delineate the regulatory process from proteins to metabolism and reveal the upstream and downstream regulatory pathways of key proteins and metabolites, we conducted a joint analysis of metabolomics and proteomics. The analysis was divided into two parts: joint analysis based on the expression level and joint analysis on the pathway level. At the expression level, we ranked the differential metabolites and differential proteins according to the *P*-value and selected the top 20 metabolites and proteins for correlation analysis (Supplementary Fig. 3). The results revealed a correlation between the differential metabolites and differential proteins. Also, at the content level, the metabolomics data and proteomics data were strongly correlated. At the pathway level, we screened for common pathways enriched by metabolomics and proteomics (Fig. 5A); among them, 44 pathways were enriched by metabolomics, 252 pathways were enriched by proteomics, and 29 pathways were common to both (Fig. 5B).

In TCM, SZ is a complex black box. Its manifestation is a series of symptoms, but its intrinsic etiology may involve multiple pathways, tissues, and organs. Therefore, to explain the diverse pathogenesis of SZ, we obtained differential protein/metabolite-common pathway-symptom flow maps based on the biological significance of the pathways and their correlation with SZ symptoms (See supplementary Table 4 for references) and retained the 18 pathways common to the differential metabolites and differential proteins (Fig. 5C). The 18 common pathways were pathogenic *Escherichia coli* infection, rheumatoid arthritis, arachidonic acid metabolism, Fc gamma R-mediated phagocytosis, oxytocin signaling pathway, GnRH signaling pathway, the C-type lectin receptor signaling pathway, serotonergic synapses, thermogenesis, the regulation of lipolysis in adipocytes, renin secretion, the peroxisome proliferator-activated receptor (PPAR) signaling pathway, bile secretion, inflammatory mediator regulation of TRP channels, circadian entrainment, vitamin digestion and absorption, fat digestion and absorption, and taste transduction. The results of the flow map revealed the pathways associated with SZ symptoms and could be traced upstream to the metabolites/proteins causing changes in the pathways, which revealed the molecular mechanism underlying SZ.

### Pathogenic Escherichia coli infection, rheumatoid arthritis, PPAR signaling pathway, bile secretion, GnRH signaling pathway, fat digestion and absorption are the key pathways associated with the main symptoms of SZ

**Fig. 6:**
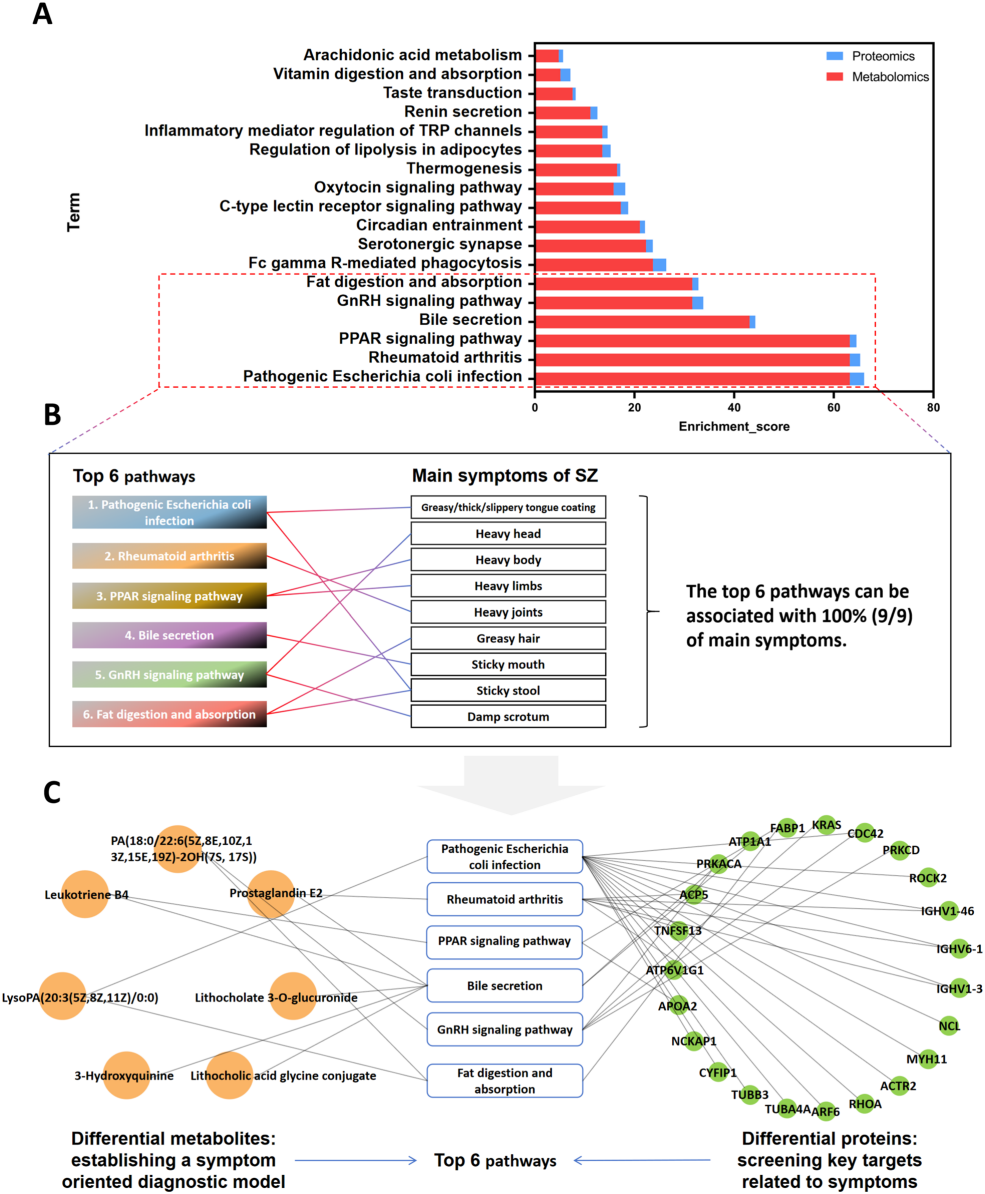
Screening of key pathways associated with SZ main symptoms. **A** Ranking map of enrichment scores of 18 common pathways got six key pathways. **B** Association map of the top six common pathways with main symptoms. **C** Differential metabolites and differential proteins associated with the top 6 common pathways

The diagnostic symptoms of SZ are divided into the main symptoms and secondary symptoms (Table 1). The main symptoms are common characteristics of SZ, and the secondary symptoms are individualized performance indicators. We identified the common characteristics of SZ and screened potential biomarkers for its diagnosis. Therefore, this study focused on optimizing the common pathways based on the main symptoms and screening the key pathways that can represent the common characteristics of SZ. By ranking the enrichment scores of the 18 common pathways (Fig. 6A), we found that the top six pathways with enrichment scores were pathogenic *Escherichia coli* infection, rheumatoid arthritis, PPAR signaling pathway, bile secretion, GnRH signaling pathway, and fat digestion and absorption. Based on the association map (Fig. 5C), the corresponding relationship between the top six pathways and the main symptoms (Fig. 6B) was determined, and the results revealed that the top six pathways were associated with 100% (9/9) of the main symptoms.

Next, the differential metabolites and differential proteins in the top six pathways were extracted (Fig. 6C). There were seven differential metabolites, which included LysoPA (20:3(5Z,8Z,11Z)/0:0), prostaglandin E2, leukotriene B4, lithocholate 3-O-glucuronide, 3-hydroxyquinine, lithocholic acid glycine conjugate, and PA(18:0/22:6(5Z,8E,10Z,13Z,15E,19Z)-2OH(7S, 17S)). Seven differential metabolites were associated with nine main symptoms through the top six pathways, and these metabolites were used to construct the diagnostic model. There were 23 differential proteins, including PRKACA, ATP1A1, FABP1, KRAS, CDC42, PRKCD, ROCK2, IGHV1-46, IGHV6-1, IGHV1-3, NCL, MYH11, ACTR2, RHOA, ARF6, TUBA4A, TUBB3, CYFIP1, NCKAP1, APOA2, ATP6V1G1, TNFSF13, and ACP5. The 23 differential proteins were associated with nine main symptoms through the top six pathways, and these proteins were used for key target screening.

### PA(18:0/22:6(5Z,8E,10Z,13Z,15E,19Z)-2OH(7S, 17S), lithocholic acid glycine conjugate, LysoPA(20:3(5Z,8Z,11Z)/0:0), prostaglandin E2, leukotriene B4, 3-hydroxyquinine, and lithocholate 3-O-glucuronide are associated with the main symptoms and constitute a stable diagnostic model for SZ

**Fig. 7:**
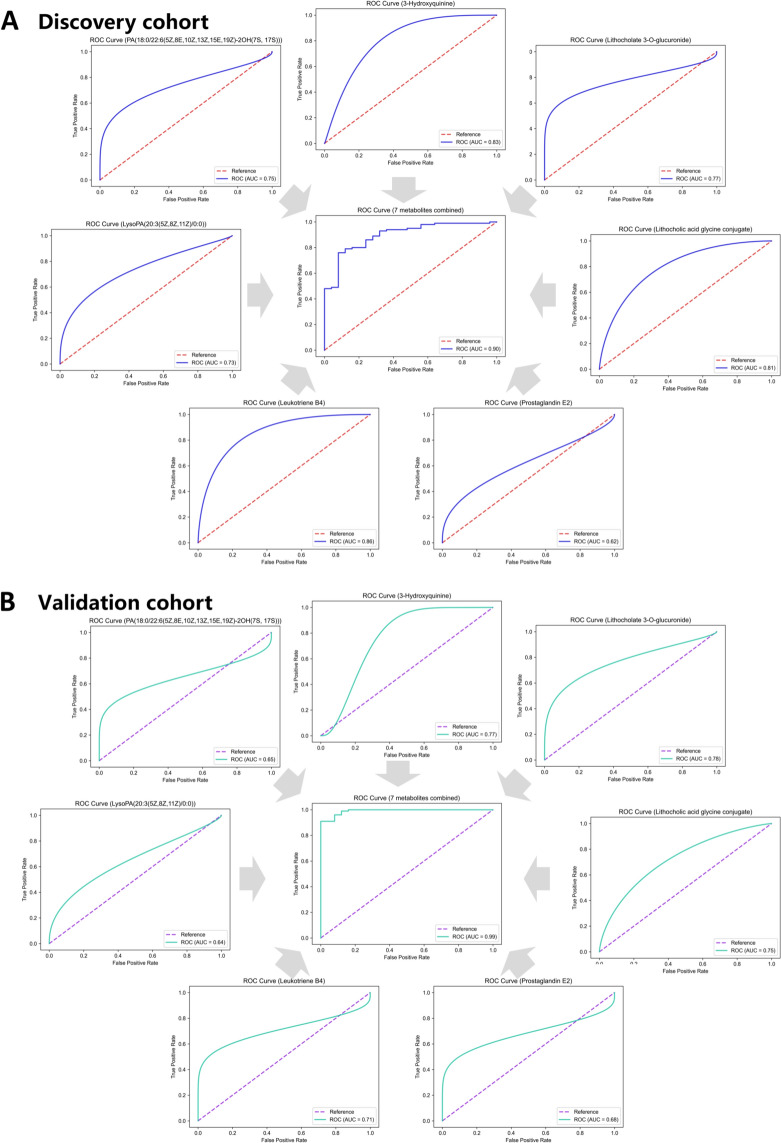
ROC analysis of differential metabolites within the six key pathways. **A** LysoPA(20:3(5Z,8Z,11Z)/0:0), Prostaglandin E2, Leukotriene B4, Lithocholate 3-O-glucuronide, 3-Hydroxyquinine, Lithocholic acid glycine conjugate, PA(18:0/22:6(5Z,8E,10Z,13Z,15E,19Z)-2OH(7S, 17S) single indicator ROC (AUC_Max_ = 0.86) and 7 indicators combined ROC (AUC = 0.90) in discovery cohort. **B** LysoPA(20:3(5Z,8Z,11Z)/0:0), Prostaglandin E2, Leukotriene B4, Lithocholate 3-O-glucuronide, 3-Hydroxyquinine, Lithocholic acid glycine conjugate, PA(18:0/22:6(5Z,8E,10Z,13Z,15E,19Z)-2OH(7S, 17S) single indicator ROC (AUC_Max_ = 0.78) and 7 indicators combined ROC (AUC = 0.99) in validation cohort

*Shi Zheng* (SZ) is an aggregate of symptoms, and the biomarkers of a single indicator are not sufficient to represent the overall changes in SZ. Therefore, to study SZ, one or two diagnostic indicators were selected for each main symptom, and then, all diagnostic indicators were fitted to construct a joint diagnostic model, which could more accurately represent the overall changes in SZ. LysoPA (20:3(5Z,8Z,11Z)/0:0), prostaglandin E2, leukotriene B4, lithocholate 3-O-glucuronide, 3-hydroxyquinine, lithocholic acid glycine conjugate, and PA(18:0/22:6(5Z,8E,10Z,13Z,15E,19Z)-2OH(7S, 17S) were found to be associated with all the main symptoms through pathogenic *Escherichia coli* infection, rheumatoid arthritis, PPAR signaling pathway, bile secretion, GnRH signaling pathway, and fat digestion and absorption pathways. Thus, these seven indicators were selected to establish a combined diagnostic model. The ROC curve revealed that in the discovery cohort, the AUC of the combined diagnosis was 0.9, and the maximum AUC of a single indicator was 0.86 (Fig. 7A). In the validation cohort, the AUC for the combined diagnosis was 0.99, and the maximum AUC for a single indicator was 0.78 (Fig. 7B).

### RHOA, TNFSF13, PRKCD, APOA2, ATP1A1, and FABP1 are associated with the main symptoms and identified as potential therapeutic targets of SZ

**Fig. 8:**
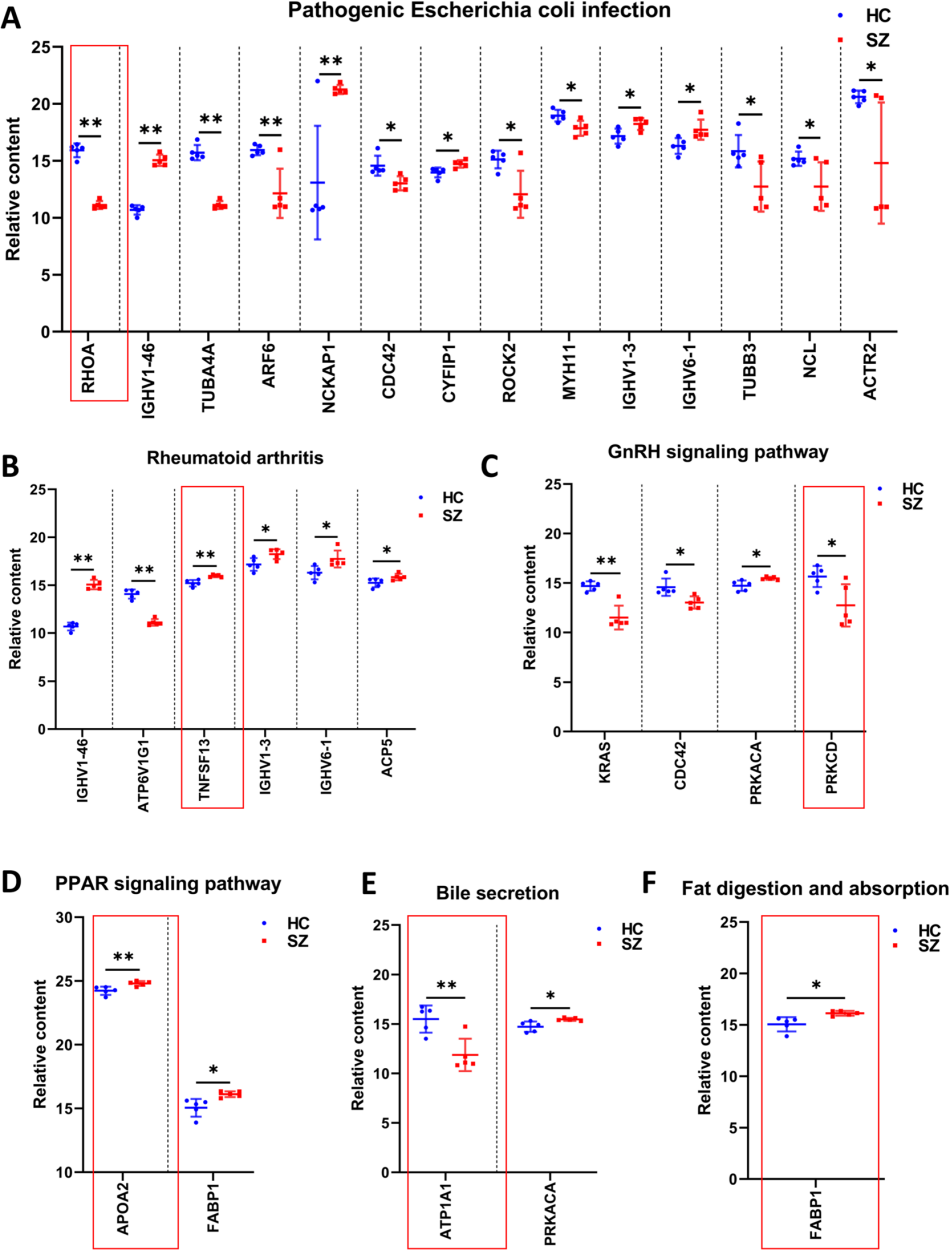
Screening of protein targets within six key pathways (n = 5). **A** Comparison of relative protein content within the Pathogenic *Escherichia coli* infection pathway. **B** Comparison of relative protein content within the Rheumatoid arthritis pathway. **C** Comparison of relative protein content within the GnRH signaling pathway. **D** Comparison of relative protein content within the PPAR signaling pathway. **E** Comparison of relative protein content within the Bile secretion pathway. **F** Comparison of relative protein content within the Fat digestion and absorption pathway. Data were analyzed by unpaired t test, and the significant difference is indicated by *P* < 0.05, *P < 0.05, **P < 0.01

The six key pathways (pathogenic *Escherichia coli* infection, rheumatoid arthritis, PPAR signaling pathway, bile secretion, GnRH signaling pathway, and fat digestion and absorption) contained 23 differentially expressed proteins. Among them, 14 were involved in the pathogenic *Escherichia coli* infection pathway (Fig. 8A), six in the rheumatoid arthritis pathway (Fig. 8B), two in the PPAR signaling pathway (Fig. 8D), two in the bile secretion pathway (Fig. 8E), four in the GnRH signaling pathway (Fig. 8C), and one in the fat digestion and absorption pathway (Fig. 8F). Differential proteins in each pathway were sorted according to the *P*-value from the smallest to the largest, and six key proteins were screened by combining the *P*-value and the position and biological significance of the proteins in the pathway (Supplementary Figs. 4–7).

When screening the target proteins according to the position in the pathway, the upstream proteins with a wider impact on the pathway should be selected. In addition, the change trend of protein content is consistent with the trend of pathway pathogenicity. For example, in the pathogenic *Escherichia coli* pathway, pathogenic bacteria can produce virulence factors that target RhOA protein and damage the activity of RhOA protein, thereby destroying the host epithelial/endothelial barrier, paralysing the migration and phagocytosis of immune cells, invading and spreading into epithelial cells, and inhibiting cell division [14, 15]. This suggests that RHOA plays an important role in the pathogenesis of *Escherichia coli*, and the development of drugs targeting RHOA protein for the treatment of *Escherichia coli* infection is of great potential. In addition, RHOA was decreased in the SZ group, which was in line with the trend of pathogenesis, so RHOA was selected as the target protein of pathogenic Escherichia coli infection pathway. The protein targets of the other 5 key pathways were screened by the same method, and the rheumatoid arthritis pathway was TNFSF13, the PPAR signaling pathway was APOA2, the bile secretion pathway was ATP1A1, the GnRH signaling pathway was PRKCD, the fat digestion and absorption pathway was FABP1. Six key targets were associated with symptoms through the corresponding pathways, which can be used as a reference for the precise treatment of SZ.

## Discussion

Previous studies on TCM *Zheng* focused on the background of the same disease [10, 16]. However, when the disease background changes, the biomarkers of the same *Zheng* become inconsistent [17], and the limitation of disease background may interfere with the study of pathological mechanism of diseases. Therefore, without a fixed disease background, we selected patients with SZ and healthy individuals from the Guangzhou No. 11 People's Hospital and collected serum for metabolomics and proteomics analysis. We performed differential analysis and enrichment analysis based on metabolomics and proteomics data, respectively, and identified 18 common pathways that could be associated with SZ symptoms. Our findings may provide a reference for the diagnosis and treatment of the symptoms of SZ. To focus on the key pathways of SZ, we selected six pathways (pathogenic *Escherichia coli* infection, rheumatoid arthritis, PPAR signaling pathway, bile secretion, GnRH signaling pathway, fat digestion, and absorption) that represent the common characteristics of SZ from 18 common pathways based on the enrichment scores of the pathways and the degree of association with the main symptoms of SZ.

We associated pathways with symptoms through the above-mentioned method, and the differential metabolites and proteins in these pathways were specific factors for the abnormal biological function of the pathway. The seven differential metabolites [LysoPA(20:3(5Z,8Z,11Z)/0:0), prostaglandin E2, leukotriene B4, lithocholate 3-O-glucuronide, 3-hydroxyquinine, lithocholic acid glycine conjugate, and PA(18:0/22:6(5Z,8E,10Z,13Z,15E,19Z)-2OH(7S, 17S))] were associated with nine main symptoms of SZ through six key pathways (Fig. 6B, C), and each symptom had a direct corresponding metabolite. Therefore, the combined diagnostic model with seven differential metabolites can monitor SZ holistically and with TCM characteristics. These results also showed that the AUC of the combined diagnosis was greater. Additionally, the combined diagnostic model designed in combination with symptoms was more advantageous than earlier, more extensive biomarker screening methods (Supplementary Fig. 2A–F). In total, 23 differential proteins were associated with nine main symptoms of SZ through six key pathways (Fig. 6B, C), and abnormalities in these six pathways were responsible for the occurrence of SZ. In this study, six key targets, including RHOA, TNFSF13, APOA2, ATP1A1, PRKCD, and FABP1, were screened from the six key pathways according to the *P* value and KEGG map. These six proteins constituted a potential target group for reversing pathway abnormalities and improving symptoms.

*Escherichia coli* is a foodborne pathogen that is present in the environment, and its proportion in the gut is closely related to the human immune environment and intake [18]. Severe infections caused by *Escherichia coli* often present with a range of gastrointestinal symptoms, such as severe abdominal pain, diarrhea, and vomiting. These infections can decrease the abundance of beneficial intestinal flora and affect the intestinal immune microcirculation [19, 20]. Studies have shown that while mild *Escherichia coli* infections do not cause noticeable symptoms, they can still cause problems such as abnormal stool shedding and abnormal tongue coating [20, 21]. This finding also indicates the association between the pathogenic *Escherichia coli* infection pathway and the main symptoms of SZ. Many researchers in China have used *Escherichia coli* to construct SZ models, and *Escherichia coli* is considered to be one of the environmental inducers of SZ, which is also consistent with our study [22–24]. In addition, the outer membrane of *Escherichia coli* is asymmetric, with the inner leaflet containing glycerophospholipid (GPL) and the outer leaflet containing lipopolysaccharide (LPS) to resist external stress. LysoPA(20:3(5Z,8Z,11Z)/0:0) is a lysophosphatidic acid, a precursor of glycerophosphatide synthesis, which also provides theoretical support for LysoPA(20:3(5Z,8Z,11Z)/0:0) as a marker of Escherichia coli SZ invasion [25]. RHOA is a Rho GTPases, which can promote the reorganization of actin cytoskeleton, and regulate the shape, attachment and movement of cells. Studies have shown that enteropathogenic *Escherichia coli* can inhibit Rho GTPases to cause desmosomes disturbance, which promotes virus colonization and toxicity [26].

Rheumatoid arthritis is a chronic autoimmune joint disease in which persistent inflammation damages bones and joints [27]. Therefore, the rheumatoid arthritis pathway is associated with the “heavy joints” of symptoms. Prostaglandin E2 is a small molecule lipid active substance, which is related to a variety of physiological processes such as inflammation and pain response, and is the main mediator of rheumatoid arthritis [28]. TNFSF13 is a member of the tumor necrosis factor (TNF) ligand family. It is expressed in a variety of immune cells, can promote the maturation and differentiation of B cells, plays an important role in the immune response, and can affect autoimmunity and participate in rheumatoid arthritis [29]. PPAR is an important regulator of energy metabolism and is highly expressed in high-energy-consuming tissues such as the fat, liver, and muscle [30]. PPARα mainly regulates the transport and oxidation of fatty acids [31], PPARβ participates in the oxidation of fatty acids and glucose uptake [32], and PPARγ regulates the storage of fatty acids and can improve insulin sensitivity [33]. These three isoforms of PPAR are highly important for fatty acid and glucose homeostasis. When the PPAR pathway is abnormal, skeletal muscle functions that require high levels of energy are affected, which corresponds to the “heavy body” and “heavy limbs” associated with SZ symptoms. Additionally, the biological significance of the first two pathways mentioned above suggests that the immune function of SZ patients may be abnormal, further weakening the body, resulting in an “external pathogenic factor attack” and “internal immune disorder causing disease”. PPAR is also expressed in immune cells, and its role in regulating energy metabolism strongly influences immune cell differentiation and fate [34]. Leukotriene B4 is a leukotriene related to inflammatory response, which can activate PPAR [35], participate in APOA2 mediated lipid transport and regulate energy metabolism [36].

Bile acid metabolism is related to the occurrence of Sjogren’s syndrome, and some bile acids can reverse the reduction in saliva secretion caused by Sjogren’s syndrome [37, 38]. Therefore, the bile secretion pathway is associated with “sticky mouth”. Additionally, abnormal lipid digestion can lead to an increase in the water content of feces, thus increasing its viscosity [39–41]. Abnormal absorption of lipids also affects the secretion of sebaceous glands, resulting in greasy hair [42]. Therefore, fat digestion and absorption pathways are associated with “greasy hair” and “sticky stool”. Bile secretion and fat digestion and absorption pathways regulate lipid digestion and absorption. ATP1A1 is a membrane protein responsible for establishing and maintaining the electrochemical gradients of Na and K ions across the plasma membrane [43], and can affect the production and secretion of bile acids by regulating osmotic pressure, and then affect the expression of metabolites such as 3-hydroxyquinine and lithocholic acid glycine conjugate. FABP1 is a fatty acid binding protein, which is involved in the metabolism of a variety of fatty acids. In addition, FABP1 can combine with bile acids and affect the content of phosphatidic acid and lysophosphatidic acid [44]. Gonadotropin-releasing hormone (GnRH) can affect the scrotum and testis by regulating the secretion of gonadotropins. It can also promote anxiety and depression, leading to dizziness, low mood, and other changes [45, 46]. Therefore, the GnRH signaling pathway is associated with “damp scrotum” and “heavy head”. Moreover, PRKCD can regulate the production of phosphatidic acid and promote the secretion of gonadotropin (Supplementary Fig. 6A). In conclusion, the occurrence of SZ is a complex process involving a variety of pathological mechanisms. The contents of this study can provide different levels of reference for the diagnosis and treatment of SZ. In addition, although there is less direct evidence for the treatment of SZ by regulating the target protein, a number of studies have shown that the anti-dampness traditional Chinese medicine has the effect of inhibiting the activity of *Escherichia coli* [47–49], which is consistent with the part of our study. This study is an exploration of a new model for the study of SZ in TCM, and a large number of studies are still needed to further verify and optimize the diagnosis and treatment model.

To summarize, this study investigated the pathways associated with SZ using symptoms as the starting point. A multi-indicator diagnostic model with TCM characteristics was designed, and a group of potential target clusters for SZ treatment was identified. Our findings provided an important reference for the discovery and treatment of SZ and investigated new paradigms for constructing TCM *Zheng* diagnosis and treatment models.

## Conclusion

The occurrence and development of SZ realted with a multi-indicator diagnostic model of SZ reflect TCM characteristics are established with LysoPA (20:3(5Z,8Z,11Z)/0:0), Prostaglandin E2, Leukotriene B4, Lithocholate 3-O-glucuronide, 3-Hydroxyquinine, Lithocholic acid glycine conjugate and PA(18:0/22:6(5Z,8E,10Z,13Z,15E,19Z)-2OH(7S, 17S) as diagnostic indicators, Which associated with 6 key pathways, such as Pathogenic Escherichia coli infection, Rheumatoid arthritis, PPAR signaling pathway, Bile secretion, GnRH signaling pathway, Fat digestion and absorption. RHOA, TNFSF13, APOA2, ATP1A1, PRKCD and FABP1 proteins were the potential target for SZ treatment.

## Supplementary Information

Supplementary Material 1.

## Data Availability

Data will be provided on reasonable request.

## Abbreviations

SZ: *Shi Zheng/*Syndrome of dampness
TCM: Traditional Chinese Medicine
HC: Healthy control
Rt: Retention time
HMDB: Human Metabolome Database
PPAR: Peroxisome proliferator-activated receptor
GnRH: Gonadotropin-releasing hormone
ACP1: Acid phosphatase 1
ACP5: Acid phosphatase 5
ACTR2: Actin related protein 2
APOA2: Apolipoprotein A2
ARF6: ADP ribosylation factor 6
ATP1A1: ATPase Na^+^/K^+^transporting subunit alpha 1
ATP6V1G1: ATPase H^+^ transporting V1 subunit G1
C1QA: Complement C1q A chain
C1QB: Complement C1q B chain
C1QC: Complement C1q C chain
C1R: Complement C1r
C1S: Complement C1s
C2: Complement C2
C4A: Complement C4A (Chido/Rodgers blood group)
CAPZA1: Capping actin protein of muscle Z-line subunit alpha 1
CAPZB: Capping actin protein of muscle Z-line subunit beta
CDC42: Cell division cycle 42
CDH1: Cadherin 1
CFB: Complement factor B
CFI: Complement factor I
CYFIP1: Cytoplasmic FMR1 interacting protein 1
DYNLL1: Dynein light chain LC8-type 1
EGF: Epidermal growth factor
F13B: Coagulation factor XIII B chain
FABP1: Fatty acid binding protein 1
FLNA: Filamin A
FYB1: FYN binding protein 1
GNAI2: G protein subunit alpha i2
HSP90AB2P: Heat shock protein 90 alpha family class B member 2, pseudogene
HSPA5: Heat shock protein family A (Hsp70) member 5
IGHV1-3: Immunoglobulin heavy variable 1–3
IGHV1-46: Immunoglobulin heavy variable 1–46
IGHV6-1: Immunoglobulin heavy variable 6–1
KRAS: KRAS proto-oncogene, GTPase
MYH11: Myosin heavy chain 11
MYL12B: Myosin light chain 12B
MYL4: Myosin light chain 4
MYL6: Myosin light chain 6
NCKAP1: NCK associated protein 1
NCL: Nucleolin
PFN1: Profilin 1
PRKACA: Protein kinase cAMP-activated catalytic subunit alpha
PRKCD: Protein kinase C delta
RAB5A: RAB5A, member RAS oncogene family
RAB7A: RAB7A, member RAS oncogene family
RACK1: Receptor for activated C kinase 1
RHOA: Ras homolog family member A
RHOG: Ras homolog family member G
ROCK2: Rho associated coiled-coil containing protein kinase 2
RPL12: Ribosomal protein L12
RPL15: Ribosomal protein L15
RPL30: Ribosomal protein L30
RPL4: Ribosomal protein L4
RPL7A: Ribosomal protein L7a
RPS14: Ribosomal protein S14
RPS16: Ribosomal protein S16
RPS20: Ribosomal protein S20
RPS21: Ribosomal protein S21
RPS4X: Ribosomal protein S4 X-linked
SERPING1: Serpin family G member 1
SIPA1: Signal-induced proliferation-associated 1
TLN1: Talin 1
TNFSF13: TNF superfamily member 13
TPM2: Tropomyosin 2
TPM3: Tropomyosin 3
TUBA4A: Tubulin alpha 4a
TUBB3: Tubulin beta 3 class III
MYH11: Myosin heavy chain 11

## References

[1] Rao S, Ahuja NK, Bharucha AE, Brenner DM, Chey WD, Deutsch JK, et al. Optimizing the utility of anorectal manometry for diagnosis and therapy: a roundtable review and recommendations. Clin Gastroenterol Hepatol. 2023;21(11):2727–39. 37302444 10.1016/j.cgh.2023.05.025 PMC10542660

[2] Yin C, Hinckel BB. Soft tissue lengthening for flexion dislocation of patella. Curr Rev Musculoskelet Med. 2023;16:531. 37665483 10.1007/s12178-023-09865-9 PMC10587048

[3] Bi JL, Chen J, Sun XM, Nie XL, Liu YY, Luo R, et al. The development and evaluation of a sub-health self-rating scale for university students in China. BMC Public Health. 2019;19(1):330. 30898160 10.1186/s12889-019-6650-3 PMC6429791

[4] Sun BG, Liu J. Graduated quantitative diagnosis of spleen deficiency syndrome. J Integr Tradit Western Med. 1994;14(3):135–8. 7524832

[5] Zhong S, Li J, Li L, Huang S, Yang M, Qiu H, et al. A review of diagnostic criteria and objective study of spleen-qi deficiency syndrome. Lishizhen Med Mater Med. 2021;32(02):421–3.

[6] Sun H, Zhang A, Wang X. Potential role of metabolomic approaches for Chinese medicine syndromes and herbal medicine. Phytother Res. 2012;26(10):1466–71. 22422429 10.1002/ptr.4613

[7] Zhang AH, Sun H, Qiu S, Wang XJ. Recent highlights of metabolomics in Chinese medicine syndrome research. Evid Based Complement Alternat Med. 2013;2013: 402159. 24302964 10.1155/2013/402159 PMC3834606

[8] Wang X, Zhang A, Han Y, Wang P, Sun H, Song G, et al. Urine metabolomics analysis for biomarker discovery and detection of jaundice syndrome in patients with liver disease. Mol Cell Proteomics. 2012;11(8):370–80. 22505723 10.1074/mcp.M111.016006 PMC3412968

[9] Zhang A, Sun H, Han Y, Yuan Y, Wang P, Song G, et al. Exploratory urinary metabolic biomarkers and pathways using UPLC-Q-TOF-HDMS coupled with pattern recognition approach. Analyst. 2012;137(18):4200–8. 22852134 10.1039/c2an35780a

[10] Jiang N, Liu HF, Li SD, Zhou WX, Zhang YX, Zhang Q, et al. An integrated metabonomic and proteomic study on kidney-yin deficiency syndrome patients with diabetes mellitus in China. Acta Pharmacol Sin. 2015;36(6):689–98. 25937635 10.1038/aps.2014.169 PMC4594178

[11] Zhao LL, Qiu XJ, Wang WB, Li RM, Wang DS. NMR metabolomics and random forests models to identify potential plasma biomarkers of blood stasis syndrome with coronary heart disease patients. Front Physiol. 2019;10:1109. 31551804 10.3389/fphys.2019.01109 PMC6738169

[12] Li Q, Wu W, Ai Z, Zhou L, Liu S, Yang X. Research on the diagnostic criteria for dampness syndrome based on consensus method. Moderniz Tradit Chin Med Mater Med World Sci Technol. 2024;26(06):1660–7.

[13] Fu-Qin K, Xue-Yin C, Geng-Hang C, LiHong Y, Shao-Nan L, Y-Han H, et al. Study on the correlation between dampness syndrome and body composition in overweight/obese population. J Guangzhou Univ Tradit Chin Med. 2023;40(08):1857–62.

[14] Lemichez E, Aktories K. Hijacking of Rho GTPases during bacterial infection. Exp Cell Res. 2013;319(15):2329–36. 23648569 10.1016/j.yexcr.2013.04.021

[15] Huelsenbeck SC, May M, Schmidt G, Genth H. Inhibition of cytokinesis by *Clostridium difficile* toxin B and cytotoxic necrotizing factors–reinforcing the critical role of RhoA in cytokinesis. Cell Motil Cytoskeleton. 2009;66(11):967–75. 19504561 10.1002/cm.20390

[16] Yang G, Zhou S, He H, Shen Z, Liu Y, Hu J, et al. Exploring the “gene-protein-metabolite” network of coronary heart disease with phlegm and blood stasis syndrome by integrated multi-omics strategy. Front Pharmacol. 2022;13:1022627. 36523490 10.3389/fphar.2022.1022627 PMC9744761

[17] Jiang M, Lu C, Zhang C, Yang J, Tan Y, Lu A, et al. Syndrome differentiation in modern research of traditional Chinese medicine. J Ethnopharmacol. 2012;140(3):634–42. 22322251 10.1016/j.jep.2012.01.033

[18] Yang SC, Lin CH, Aljuffali IA, Fang JY. Current pathogenic Escherichia coli foodborne outbreak cases and therapy development. Arch Microbiol. 2017;199(6):811–25. 28597303 10.1007/s00203-017-1393-y

[19] Mueller M, Tainter CR. Escherichia coli Infection. 2024. 33231968

[20] Malavolta M, Basso A, Giacconi R, Orlando F, Pierpaoli E, Cardelli M, et al. Recovery from mild Escherichia coli O157:H7 infection in young and aged C57BL/6 mice with intact flora estimated by fecal shedding, locomotor activity and grip strength. Comp Immunol Microbiol Infect Dis. 2019;63:1–9. 30961802 10.1016/j.cimid.2018.12.003

[21] Li X, Sun Y, Cao X, Shi Y, Xie W, Shi L. Correlation study between intestinal flora and tongue coating in patients with chronic gastritis. Chin J Integr Tradit Western Med Digest. 2021;29(11):762–8.

[22] Zhang Y, Yao W, Zhang W, Wen Y, Hua Y, Ji P, et al. Yujin powder improves large intestine dampness-heat syndrome by regulating gut microbiota and serum metabolism. Biomed Chromatogr. 2023;37(11): e5719. 37605605 10.1002/bmc.5719

[23] Yao W, Yang C, Wen Y, Zhang W, Zhang X, Ma Q, et al. Treatment effects and mechanisms of Yujin Powder on rat model of large intestine dampness-heat syndrome. J Ethnopharmacol. 2017;202:265–80. 28330724 10.1016/j.jep.2017.03.030

[24] Hua YL, Ma Q, Zhang XS, Jia YQ, Peng XT, Yao WL, et al. Pulsatilla decoction can treat the dampness-heat diarrhea rat model by regulating glycerinphospholipid metabolism based lipidomics approach. Front Pharmacol. 2020;11:197. 32194420 10.3389/fphar.2020.00197 PMC7064006

[25] Rex AN, Simpson BW, Bokinsky G, Trent MS. PlsX and PlsY: additional roles beyond glycerophospholipid synthesis in gram-negative bacteria. MBio. 2024;15(12): e296924. 10.1128/mbio.02969-24 PMC11633183 39475235

[26] Roxas JL, Monasky RC, Roxas BAP, Agellon AB, Mansoor A, Kaper JB, et al. Enteropathogenic Escherichia coli EspH-mediated Rho GTPase inhibition results in desmosomal perturbations. Cell Mol Gastroenterol Hepatol. 2018;6(2):163–80. 30003123 10.1016/j.jcmgh.2018.04.007 PMC6039986

[27] Radu AF, Bungau SG. Management of rheumatoid arthritis: an overview. Cells. 2021;10(11):2857. 34831081 10.3390/cells10112857 PMC8616326

[28] Park JY, Pillinger MH, Abramson SB. Prostaglandin E2 synthesis and secretion: the role of PGE2 synthases. Clin Immunol. 2006;119(3):229–40. 16540375 10.1016/j.clim.2006.01.016

[29] Xiao Y, Motomura S, Podack ER. APRIL (TNFSF13) regulates collagen-induced arthritis, IL-17 production and Th2 response. Eur J Immunol. 2008;38(12):3450–8. 19016524 10.1002/eji.200838640 PMC2755626

[30] Cheng HS, Tan WR, Low ZS, Marvalim C, Lee J, Tan NS. Exploration and development of PPAR modulators in health and disease: an update of clinical evidence. Int J Mol Sci. 2019;20(20):5055. 31614690 10.3390/ijms20205055 PMC6834327

[31] van Raalte DH, Li M, Pritchard PH, Wasan KM. Peroxisome proliferator-activated receptor (PPAR)-alpha: a pharmacological target with a promising future. Pharm Res. 2004;21(9):1531–8. 15497675 10.1023/b:pham.0000041444.06122.8d

[32] Magadum A, Engel FB. PPARbeta/delta: linking metabolism to regeneration. Int J Mol Sci. 2018;19(7):2013. 29996502 10.3390/ijms19072013 PMC6073704

[33] Janani C, Ranjitha KB. PPAR gamma gene—a review. Diabetes Metab Syndr. 2015;9(1):46–50. 25450819 10.1016/j.dsx.2014.09.015

[34] Christofides A, Konstantinidou E, Jani C, Boussiotis VA. The role of peroxisome proliferator-activated receptors (PPAR) in immune responses. Metabolism. 2021;114: 154338. 32791172 10.1016/j.metabol.2020.154338 PMC7736084

[35] Narala VR, Adapala RK, Suresh MV, Brock TG, Peters-Golden M, Reddy RC. Leukotriene B4 is a physiologically relevant endogenous peroxisome proliferator-activated receptor-alpha agonist. J Biol Chem. 2010;285(29):22067–74. 20400503 10.1074/jbc.M109.085118 PMC2903376

[36] Dai J, Li Y, Kametani F, Cui X, Igarashi Y, Huo J, et al. Curcumin promotes AApoAII amyloidosis and peroxisome proliferation in mice by activating the PPARalpha signaling pathway. Elife. 2021;10. 10.7554/eLife.63538 PMC7880682 33496266

[37] Castro I, Albornoz N, Aguilera S, Barrera MJ, Gonzalez S, Nunez M, et al. Aberrant MUC1 accumulation in salivary glands of Sjogren’s syndrome patients is reversed by TUDCA in vitro. Rheumatology (Oxford). 2020;59(4):742–53. 31377809 10.1093/rheumatology/kez316

[38] Li H, Zhan H, Cheng L, Huang Y, Li X, Yan S, et al. Plasma lipidomics of primary biliary cholangitis and its comparison with Sjogren’s syndrome. Front Immunol. 2023;14:1124443. 37215104 10.3389/fimmu.2023.1124443 PMC10196160

[39] Azer SA, Sankararaman S. Steatorrhea. 2024. 31082099

[40] McRorie J, Brown S, Cooper R, Givaruangsawat S, Scruggs D, Boring G. Effects of dietary fibre and olestra on regional apparent viscosity and water content of digesta residue in porcine large intestine. Aliment Pharmacol Ther. 2000;14(4):471–7. 10759627 10.1046/j.1365-2036.2000.00734.x

[41] Omer E, Chiodi C. Fat digestion and absorption: normal physiology and pathophysiology of malabsorption, including diagnostic testing. Nutr Clin Pract. 2024;39(Suppl 1):S6–16. 38429963 10.1002/ncp.11130

[42] Hirt PA, Castillo DE, Yosipovitch G, Keri JE. Skin changes in the obese patient. J Am Acad Dermatol. 2019;81(5):1037–57. 31610857 10.1016/j.jaad.2018.12.070

[43] Spontarelli K, Young VC, Sweazey R, Padro A, Lee J, Bueso T, et al. ATP1A1-linked diseases require a malfunctioning protein product from one allele. Biochim Biophys Acta Mol Cell Res. 2024;1871(1): 119572. 37659504 10.1016/j.bbamcr.2023.119572 PMC12701533

[44] Hotamisligil GS, Bernlohr DA. Metabolic functions of FABPs-mechanisms and therapeutic implications. Nat Rev Endocrinol. 2015;11(10):592–605. 26260145 10.1038/nrendo.2015.122 PMC4578711

[45] Yeo B, Toh E, Lim NE, Lee RS, Ho R, Tam W, et al. Association of benign paroxysmal positional vertigo with depression and anxiety—a systematic review and meta-analysis. Laryngoscope. 2024;134(2):526–34. 37560919 10.1002/lary.30957

[46] Gormanns P, Mueller NS, Ditzen C, Wolf S, Holsboer F, Turck CW. Phenome-transcriptome correlation unravels anxiety and depression related pathways. J Psychiatr Res. 2011;45(7):973–9. 21255794 10.1016/j.jpsychires.2010.12.010

[47] Gong H, Li S, He L, Kasimu R. Microscopic identification and in vitro activity of Agastache rugosa (Fisch. et Mey) from Xinjiang, China. BMC Complement Altern Med. 2017;17(1):95. 28173792 10.1186/s12906-017-1605-7 PMC5297021

[48] Wu Y, Lu W, Geng Y, Yu C, Sun H, Kim Y, et al. Antioxidant, antimicrobial and anti-inflammatory activities of essential oil derived from the wild rhizome of *Atractylodes macrocephala*. Chem Biodivers. 2020;17(8): e2000268. 32533626 10.1002/cbdv.202000268

[49] Soshnikova V, Kim YJ, Singh P, Huo Y, Markus J, Ahn S, et al. Cardamom fruits as a green resource for facile synthesis of gold and silver nanoparticles and their biological applications. Artif Cells Nanomed Biotechnol. 2018;46(1):108–17. 28290213 10.1080/21691401.2017.1296849

