# Supplementary material for: Multi-omics driven paradigm for construction of traditional Chinese Medicine Zheng (syndrome) diagnosis and treatment model, taking Shi Zheng (syndrome of dampness) as an example
Source: Chin Med. 2025 Mar 8;20:33. doi: 10.1186/s13020-025-01085-2 (PMC11890557; doi:10.1186/s13020-025-01085-2)
Supplement: Supplementary file 1 — Supplementary Material 1. [file 13020_2025_1085_MOESM1_ESM.docx]

**Supplementary material:**

Supplementary table 1. the elution gradient of LC-MS/MS high resolution mass spectrometry

| Time（min） | Gradient |
| --- | --- |
| 0 | 2% B |
| 45 | 22% B |
| 50 | 37% B |
| 55 | 80% B |
| 60 | 80% B |

Supplementary table 2. Parameters of proteomics raw data processing software

| Items | Para. |
| --- | --- |
| Missed cleavage | 2 |
| Precursor Qvalue cutoff | 0.01 |
| Protein Qvalue cutoff | 0.01 |
| Enzyme | Trypsin/P |
| Quantity MS-Level | MS2 |
| Fixed modification | Carbamidomethyl（C） |
| Variable modification | Oxidation（M）、Acetyl（Protein N-term） |

Supplementary table 3. 30 differential metabolite information

| NO | Name | RT(min) | m/z | Lon | Molecular | Trend |
| --- | --- | --- | --- | --- | --- | --- |
| 1 | 3-Methylsulfonyl-dde | 1.01 | 392.9094 | M-H | C_15_H_10_Cl_4_O_2_S | Up |
| 2 | Diethone | 3.22 | 252.1235 | M-H | C_13_H_19_NO_4_ | Up |
| 3 | PA(18:0/22:6(5Z,8E,10Z,13Z,15E,19Z)-2OH(7S, 17S)) | 4.47 | 779.4907 | M-H | C_43_H_73_O_10_P | Up |
| 4 | All trans decaprenyl diphosphate | 5.03 | 863.6071 | M-H | C_50_H_90_O_7_P_2_ | Up |
| 5 | 1-Octanesulfonic acid | 5.41 | 193.0892 | M-H | C_8_H_18_O_3_S | Up |
| 6 | N2-Succinyl-L-ornithine | 5.52 | 231.0979 | M-H | C_9_H_16_N_2_O_5_ | Up |
| 7 | 3-(2-Carboxyethylthio)-3-(2-(8-phenyloctyl)phenyl)propanoic acid | 5.52 | 441.2138 | M-H | C_26_H_34_O_4_S | Up |
| 8 | 1,11-Undecanedicarboxylic acid | 6.04 | 243.1596 | M-H | C_13_H_24_O_4_ | Down |
| 9 | Sampangine | 6.07 | 231.0582 | M-H | C_15_H_8_N_2_O | Up |
| 10 | 2-Hydroxyphenylacetic acid glucuronide | 6.15 | 321.1342 | M-H | C_17_H_22_O_6_ | Up |
| 11 | Lithocholate 3-O-glucuronide | 9.53 | 551.3229 | M-H | C_30_H_48_O_9_ | Up |
| 12 | Leukotriene B4 | 13.20 | 335.2252 | M-H | C_20_H_32_O_4_ | Up |
| 13 | PGP(i-12:0/PGF2alpha) | 14.00 | 843.4045 | M-H | C_38_H_70_O_16_P_2_ | Down |
| 14 | 5-Hydroxyindoleacetic acid | 4.87 | 192.0654 | M+H | C_10_H_9_NO_3_ | Up |
| 15 | SM(d18:0/PGF2alpha) | 4.87 | 803.5840 | M+H | C_43_H_83_N_2_O_9_P | Down |
| 16 | Assamicain C | 4.92 | 917.1706 | M+H | C_44_H_36_O_22_ | Down |
| 17 | 2-Octenoic acid | 5.11 | 143.1067 | M+H | C_8_H_14_O_2_ | Up |
| 18 | Hydroxyethylpromethazine | 5.55 | 330.1766 | M+H | C_19_H_25_N_2_OS+ | Up |
| 19 | LysoPA(20:3(5Z,8Z,11Z)/0:0) | 5.67 | 461.2651 | M+H | C_23_H_41_O_7_P | Up |
| 20 | (4R,5S,7R,11x)-11,12-Dihydroxy-1(10)-spirovetiven-2-one 12-glucoside | 6.26 | 415.2323 | M+H | C_21_H_34_O_8_ | Up |
| 21 | 3-Hydroxyquinine | 6.72 | 341.1855 | M+H | C_20_H_24_N_2_O_3_ | Up |
| 22 | Michellamine A | 6.87 | 757.3542 | M+H | C_46_H_48_N_2_O_8_ | Up |
| 23 | Rotundine A | 7.02 | 232.1695 | M+H | C_15_H_21_NO | Down |
| 24 | 5-(p-Methylphenyl)-5-phenylhydantoin | 7.09 | 267.1129 | M+H | C_16_H_14_N_2_O_2_ | Up |
| 25 | Melatonin glucuronide | 7.10 | 409.1616 | M+H | C_19_H_24_N_2_O_8_ | Up |
| 26 | Prostaglandin E2 | 7.18 | 353.2290 | M+H | C_20_H_32_O_5_ | Down |
| 27 | 1-(4-Methoxyphenyl)-1-penten-3-one | 7.76 | 191.1066 | M+H | C_12_H_14_O_2_ | Up |
| 28 | Lithocholic acid glycine conjugate | 8.28 | 434.3254 | M+H | C_26_H_43_NO_4_ | Down |
| 29 | Hexacosanoyl carnitine | 9.65 | 540.4978 | M+H | C_33_H_65_NO_4_ | Up |
| 30 | (8'R)-Neochrome | 13.57 | 599.4482 | M+H | C_41_H_58_O_3_ | Up |

Supplementary table 4. References for the association of pathways with symptoms

| Pathways | References |
| --- | --- |
| Pathogenic Escherichia coli infection | PMID: 24982324, PMID: 30961802, PMID: 30734438 |
| Rheumatoid arthritis | PMID: 27156434 |
| Arachidonic acid metabolism | PMID: 27498352, PMID: 30735766 |
| Fc gamma R-mediated phagocytosis | PMID: 12370376, PMID: 21755190 |
| Oxytocin signaling pathway | PMID: 34768894 |
| GnRH signaling pathway | PMID: 37075816, PMID: 37560919, PMID: 21255794 |
| C-type lectin receptor signaling pathway | PMID: 21350579 |
| Serotonergic synapse | PMID: 35864781, PMID: 17192569 |
| Thermogenesis | PMID: 37482656 |
| Regulation of lipolysis in adipocytes | PMID: 17313320 |
| Renin secretion | PMID: 37313725, PMID: 18712045, PMID: 23678024 |
| PPAR signaling pathway | PMID: 33137899 |
| Bile secretion | PMID: 7714463, PMID: 23897680, PMID: 31377809, PMID: 37215104 |
| Inflammatory mediator regulation of TRP channels | PMID: 15306801, PMID: 33085914, PMID: 36902145,  PMID: 26411771, PMID：26385481 |
| Circadian entrainment | PMID: 35512209 |
| Vitamin digestion and absorption | PMID: 24484678, PMID: 31940621 |
| Fat digestion and absorption | PMID: 29617643, PMID：38429963, PMID: 10759627, PMID: 31610857 |
| Taste transduction | PMID: 36422992 |


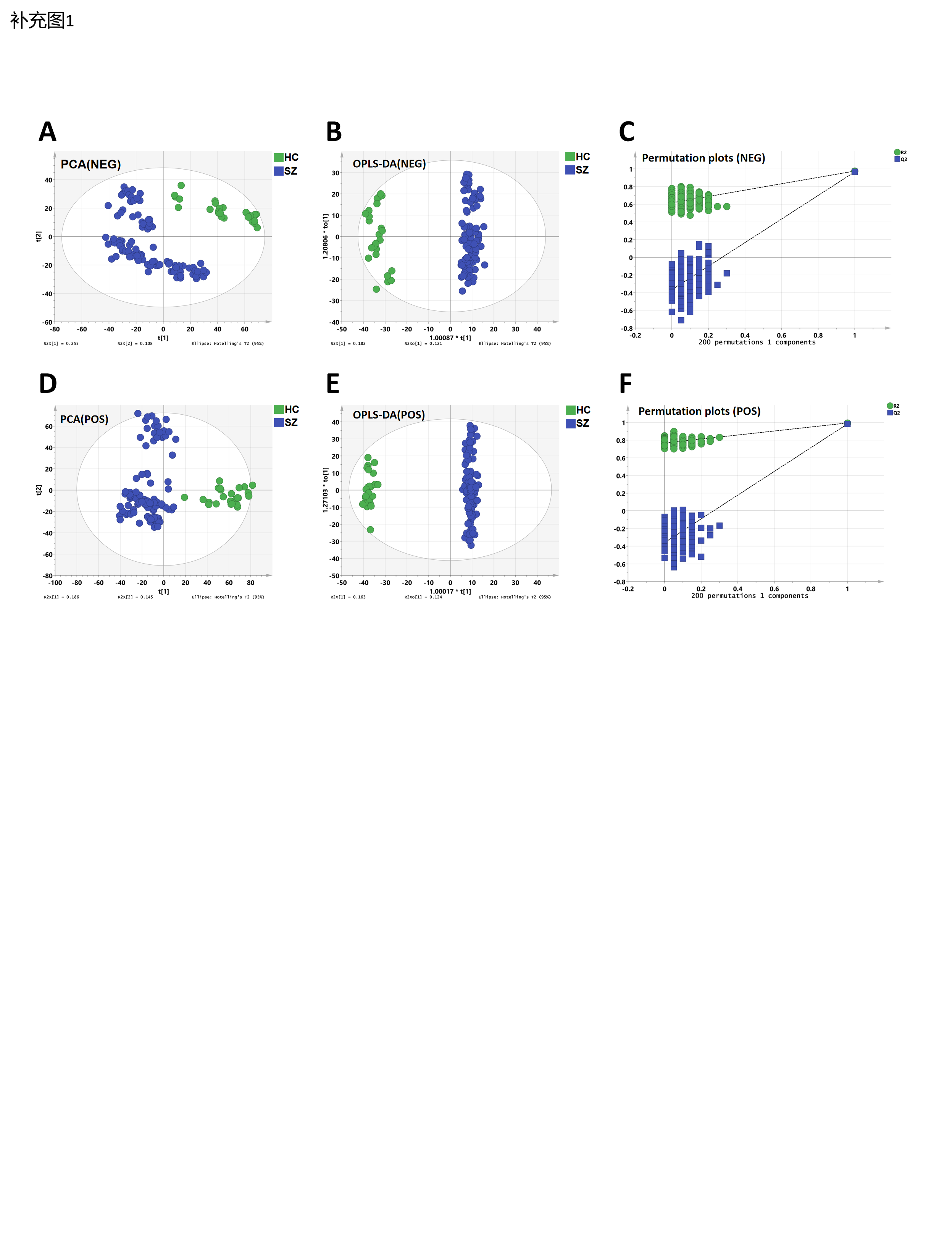


Supplementary figure 1. Analysis of differential metabolites in the serum of healthy individuals and patients with SZ in cohort 2. A, B, C: PCA, OPLS-DA scatter plots, and permutation plots of negative ion serum metabolites in healthy control and patients with SZ in cohort 2. D, E, F: PCA, OPLS-DA scatter plots and permutation plots of positive ion serum metabolites in healthy control and patients with SZ in cohort 2.


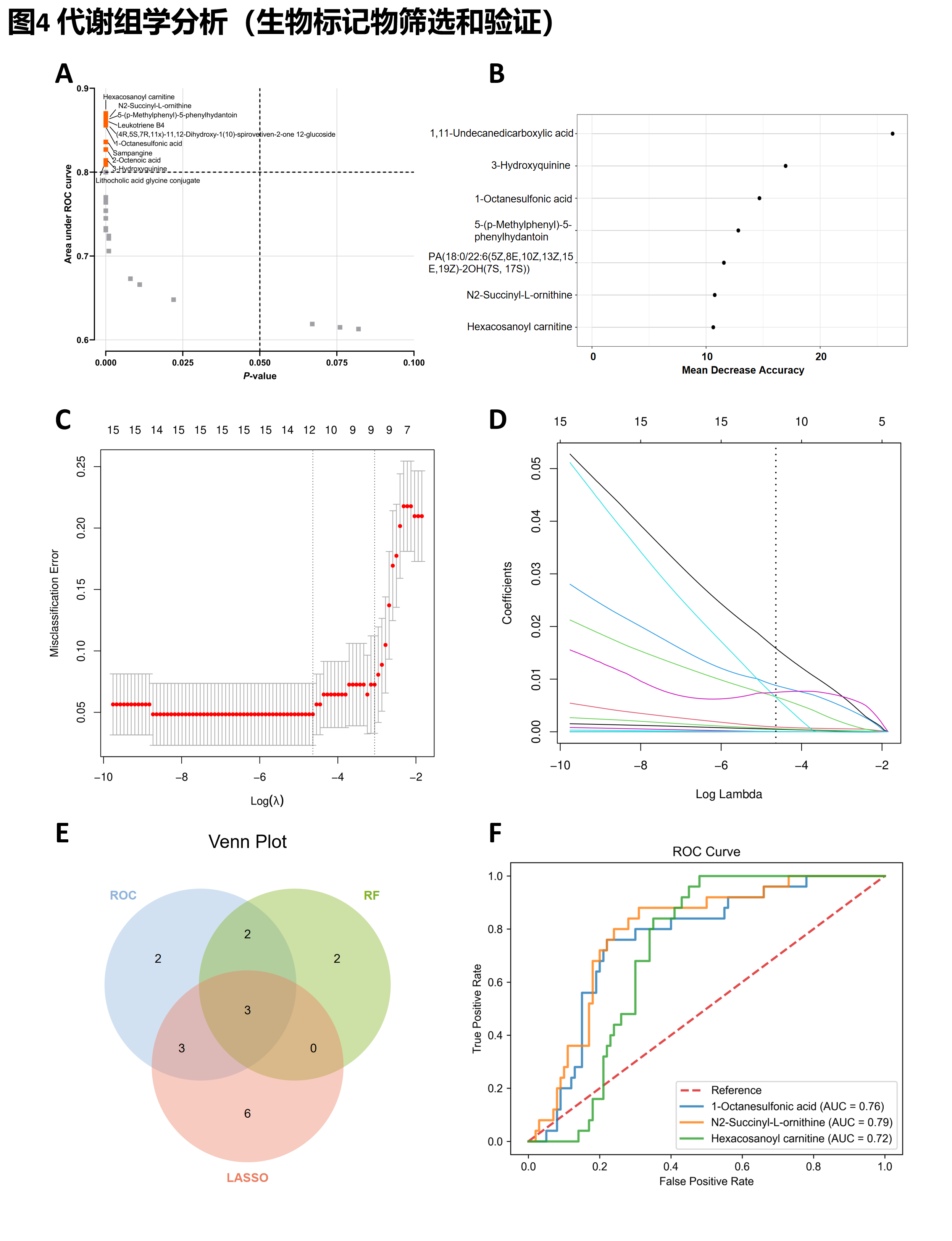


Supplementary figure 2. Screening and validation of biomarkers. A: AUC and *P* value screening chart based on ROC analysis (AUC>0.8, *P*<0.05). B: Random forest feature importance ranking plot. C: LASSO regression analysis cross validation curves. D: LASSO coefficient path diagram. E: Interactive Venn plot of three marker screening methods. F：ROC curves of three biomarkers in the validation cohort.


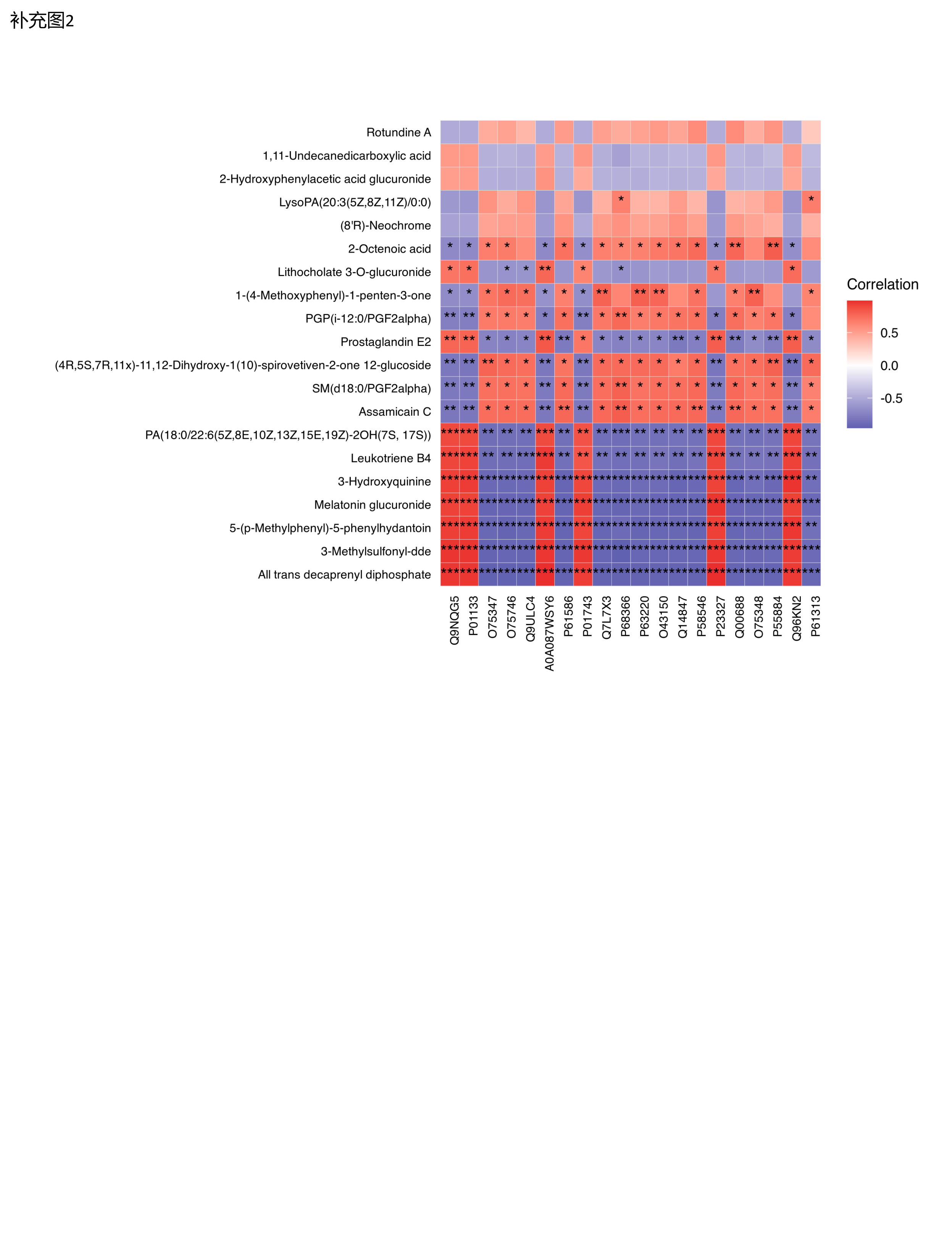


Supplementary figure 3. Correlation analysis results of the top 20 differential metabolites and top 20 differential proteins. (* *P*<0.05, ** *P*<0.01,****P*<0.001)


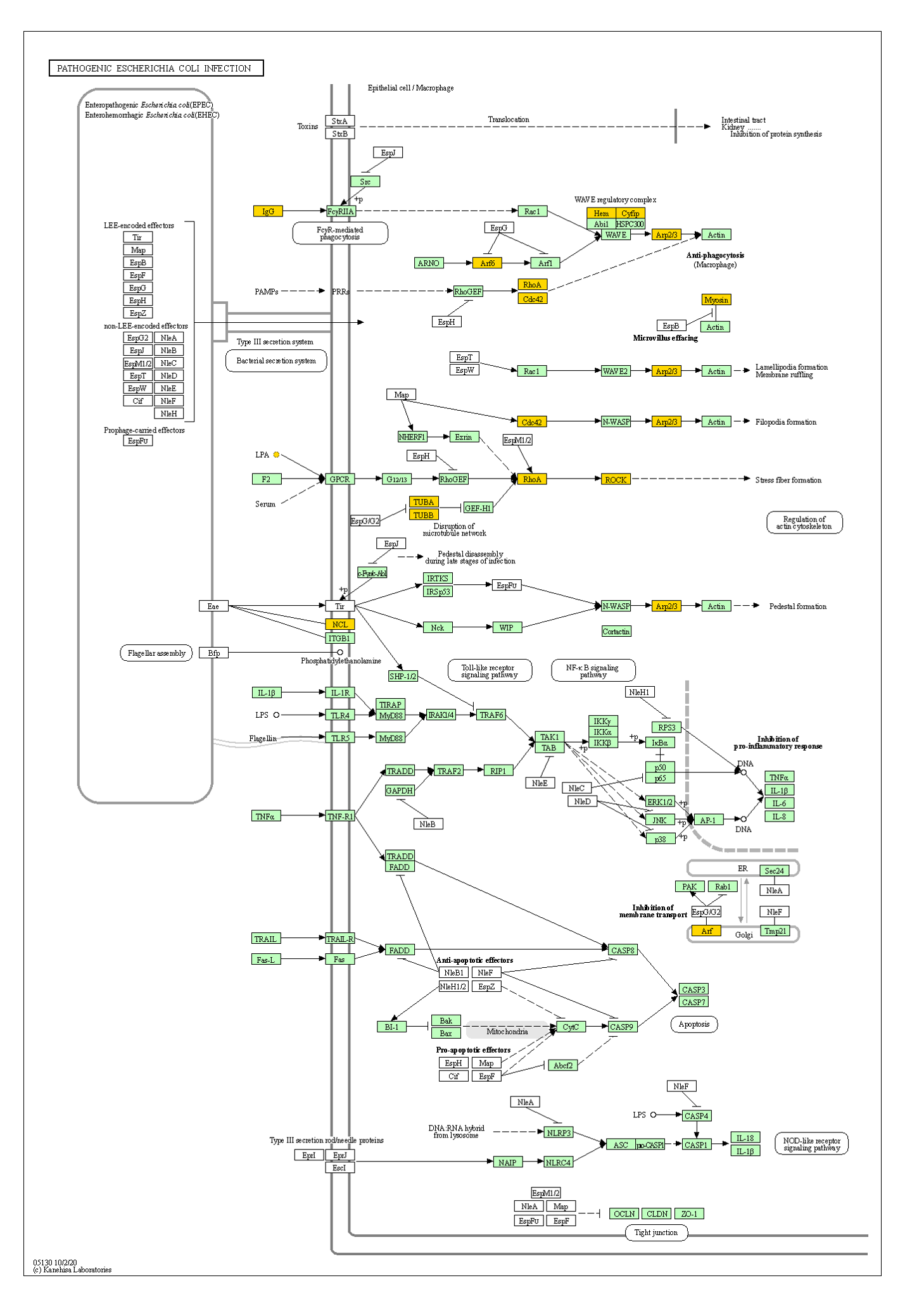


Supplementary figure 4. Distribution map of differential metabolites and proteins within pathogenic Escherichia coli infection pathway. (The yellow markers in the figure represent the differential proteins and metabolites obtained in this study)


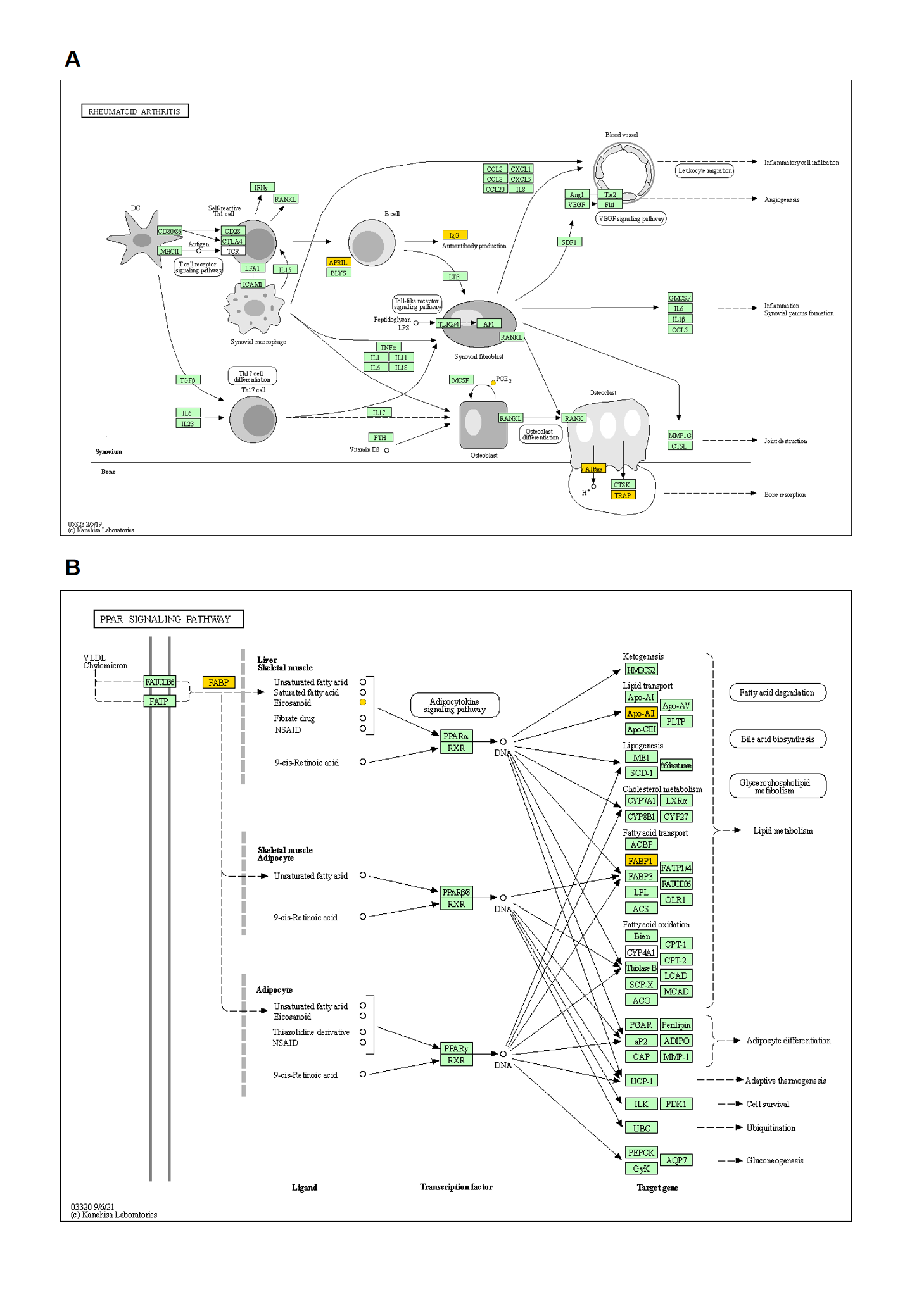


Supplementary figure 5. A: Distribution map of differential metabolites and proteins within rheumatoid arthritis pathway. B: Distribution map of differential metabolites and proteins within PPAR signaling pathway. (The yellow markers in the figure represent the differential proteins and metabolites obtained in this study)


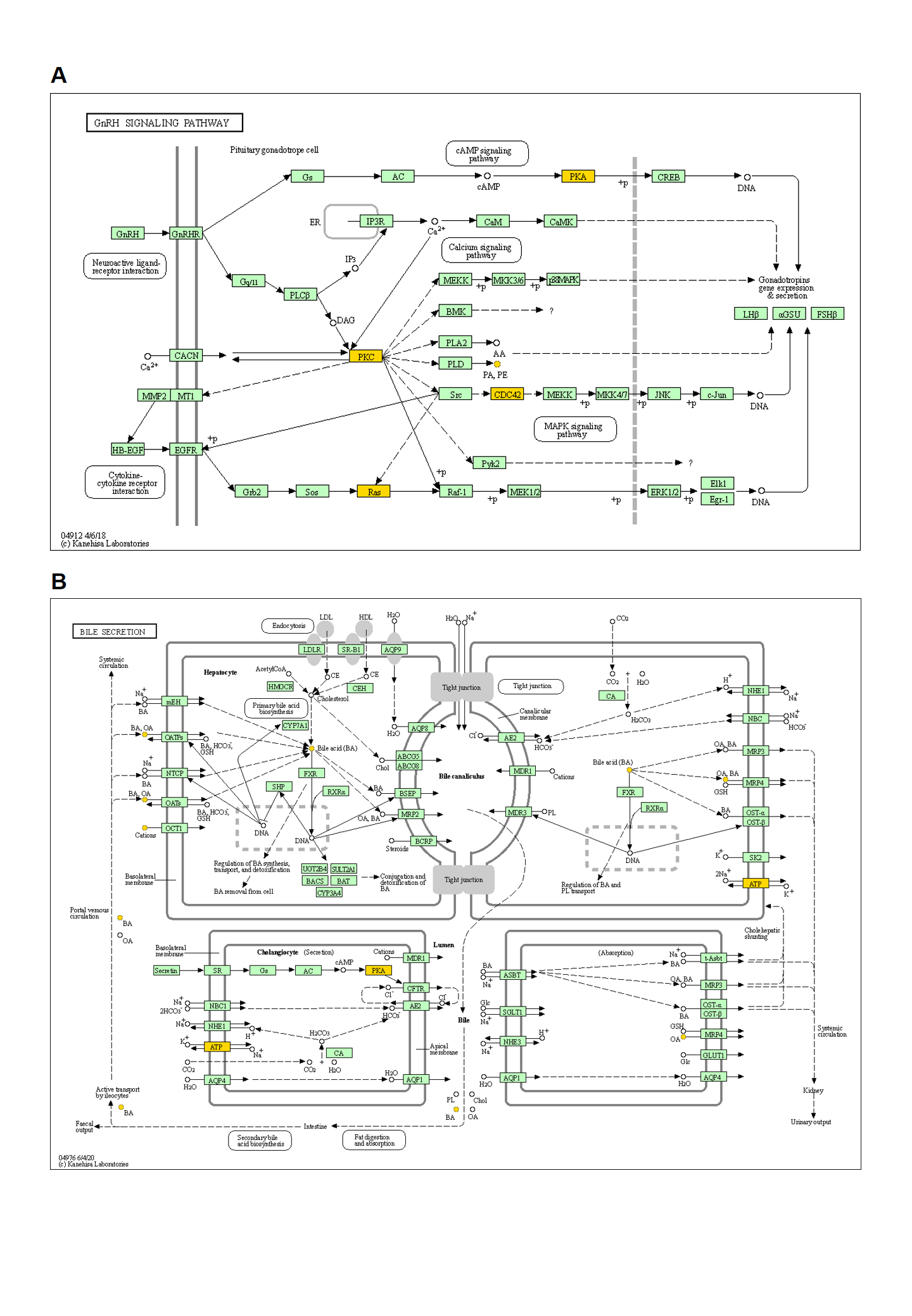


Supplementary figure 6. A: Distribution map of differential metabolites and proteins within GnRH signaling pathway. B: Distribution map of differential metabolites and proteins within bile secretion pathway. (The yellow markers in the figure represent the differential proteins and metabolites obtained in this study)


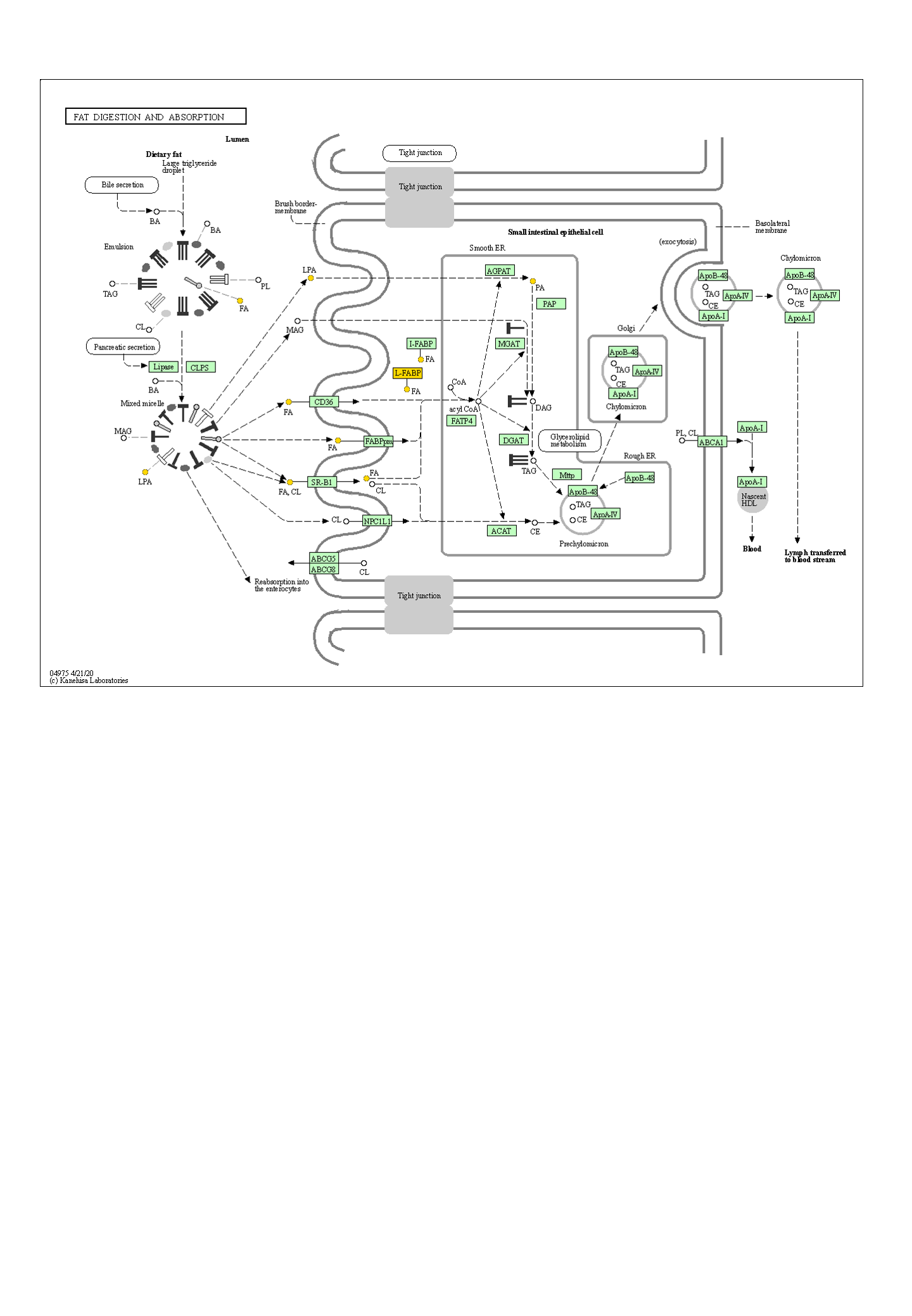


Supplementary figure 7. Distribution map of differential metabolites and proteins within fat digestion and absorption pathway. (The yellow markers in the figure represent the differential proteins and metabolites obtained in this study)
